# Supplementary material for: Exploring phytochemicals as potential pharmacological inhibitors for NS1 protein of Kyasanur forest disease virus using virtual screening, molecular docking, and molecular simulation approach
Source: PeerJ. 2025 Oct 9;13:e19954. doi: 10.7717/peerj.19954 (PMC12515432; doi:10.7717/peerj.19954)
Supplement: Supplemental Information 1 [file peerj-13-19954-s001.docx]

**Table S1**. Secondary structure analysis of NS1 protein by SOPMA and STRIDE

|  | SOPMA | STRIDE |
| --- | --- | --- |
| Alpha helix(Hh) | 74 is 20.96% | 32 is 9.06% |
| Extended strand (Ee) | 78 is 22.10% | 87 is 24.64% |
| Pi helix (Ii) | 0 is 0.00% | 0 is 0.00% |
| Bend region (Ss) | 0 is 0.00% | 0 is 0.00% |
| Random coil(Cc) | 185 is 52.41% | 81 is 22.94% |
| Beta turn(Tt) | 16 is 4.53% | 125 is 35.41% |
| Beta bridge(Bb) | 0 is 0.00% | 13 is 3.68% |
| 310 helix(Gg) | 0 is 0.00% | 15 is 4.24% |
