# Supplementary material for: Exploring phytochemicals as potential pharmacological inhibitors for NS1 protein of Kyasanur forest disease virus using virtual screening, molecular docking, and molecular simulation approach
Source: PeerJ. 2025 Oct 9;13:e19954. doi: 10.7717/peerj.19954 (PMC12515432; doi:10.7717/peerj.19954)
Supplement: Supplemental Information 2 [file peerj-13-19954-s002.docx]

**Table S2**: Secondary Virtual screening of 1150 compounds with binding energy value by PyRxk software

| **Sr.No.** | **Ligand Name** | **Binding Energy (kcal/mol)** | **Sr.No.** | **Ligand Name** | **Binding Energy**  **(kcal/mol)** |
| --- | --- | --- | --- | --- | --- |
|  | IMPHY010294 | -9.8 |  | IMPHY009130 | -8 |
|  | IMPHY010989 | -9.8 |  | IMPHY009446 | -8 |
|  | IMPHY007737 | -9.7 |  | IMPHY009648 | -8 |
|  | IMPHY017577 | -9.5 |  | IMPHY010191 | -8 |
|  | IMPHY005775 | -9.4 |  | IMPHY010356 | -8 |
|  | IMPHY005976 | -9.4 |  | IMPHY010432 | -8 |
|  | IMPHY010476 | -9.4 |  | IMPHY010647 | -8 |
|  | IMPHY009003 | -9.3 |  | IMPHY010658 | -8 |
|  | IMPHY009364 | -9.3 |  | IMPHY010673 | -8 |
|  | IMPHY000317 | -9.2 |  | IMPHY010731 | -8 |
|  | IMPHY001575 | -9.2 |  | IMPHY010912 | -8 |
|  | IMPHY004140 | -9.2 |  | IMPHY010931 | -8 |
|  | IMPHY005667 | -9.2 |  | IMPHY011387 | -8 |
|  | IMPHY006318 | -9.2 |  | IMPHY011390 | -8 |
|  | IMPHY009308 | -9.2 |  | IMPHY011921 | -8 |
|  | IMPHY002477 | -9.1 |  | IMPHY011924 | -8 |
|  | IMPHY002751 | -9.1 |  | IMPHY011951 | -8 |
|  | IMPHY003078 | -9.1 |  | IMPHY011996 | -8 |
|  | IMPHY004515 | -9.1 |  | IMPHY012234 | -8 |
|  | IMPHY004849 | -9.1 |  | IMPHY012527 | -8 |
|  | IMPHY004968 | -9.1 |  | IMPHY012576 | -8 |
|  | IMPHY006882 | -9.1 |  | IMPHY012886 | -8 |
|  | IMPHY007736 | -9.1 |  | IMPHY013094 | -8 |
|  | IMPHY009067 | -9.1 |  | IMPHY013217 | -8 |
|  | IMPHY009264 | -9.1 |  | IMPHY013231 | -8 |
|  | IMPHY000366 | -9 |  | IMPHY013449 | -8 |
|  | IMPHY002011 | -9 |  | IMPHY013599 | -8 |
|  | IMPHY003352 | -9 |  | IMPHY014394 | -8 |
|  | IMPHY003563 | -9 |  | IMPHY014422 | -8 |
|  | IMPHY005266 | -9 |  | IMPHY014700 | -8 |
|  | IMPHY005785 | -9 |  | IMPHY015465 | -8 |
|  | IMPHY007735 | -9 |  | IMPHY000055 | -7.9 |
|  | IMPHY008834 | -9 |  | IMPHY000118 | -7.9 |
|  | IMPHY010508 | -9 |  | IMPHY000119 | -7.9 |
|  | IMPHY011941 | -9 |  | IMPHY000265 | -7.9 |
|  | IMPHY014146 | -9 |  | IMPHY000290 | -7.9 |
|  | IMPHY014170 | -9 |  | IMPHY000537 | -7.9 |
|  | IMPHY002121 | -8.9 |  | IMPHY000559 | -7.9 |
|  | IMPHY002382 | -8.9 |  | IMPHY000579 | -7.9 |
|  | IMPHY003529 | -8.9 |  | IMPHY000590 | -7.9 |
|  | IMPHY004739 | -8.9 |  | IMPHY000636 | -7.9 |
|  | IMPHY005293 | -8.9 |  | IMPHY001001 | -7.9 |
|  | IMPHY007957 | -8.9 |  | IMPHY001034 | -7.9 |
|  | IMPHY009244 | -8.9 |  | IMPHY001239 | -7.9 |
|  | IMPHY009438 | -8.9 |  | IMPHY001304 | -7.9 |
|  | IMPHY009786 | -8.9 |  | IMPHY001311 | -7.9 |
|  | IMPHY010466 | -8.9 |  | IMPHY001439 | -7.9 |
|  | IMPHY010666 | -8.9 |  | IMPHY001837 | -7.9 |
|  | IMPHY011945 | -8.9 |  | IMPHY001922 | -7.9 |
|  | IMPHY012275 | -8.9 |  | IMPHY001967 | -7.9 |
|  | IMPHY000661 | -8.8 |  | IMPHY002056 | -7.9 |
|  | IMPHY001281 | -8.8 |  | IMPHY002125 | -7.9 |
|  | IMPHY001343 | -8.8 |  | IMPHY002140 | -7.9 |
|  | IMPHY003143 | -8.8 |  | IMPHY002145 | -7.9 |
|  | IMPHY004035 | -8.8 |  | IMPHY002156 | -7.9 |
|  | IMPHY004082 | -8.8 |  | IMPHY002246 | -7.9 |
|  | IMPHY004287 | -8.8 |  | IMPHY002561 | -7.9 |
|  | IMPHY004657 | -8.8 |  | IMPHY002571 | -7.9 |
|  | IMPHY008030 | -8.8 |  | IMPHY002712 | -7.9 |
|  | IMPHY008557 | -8.8 |  | IMPHY002758 | -7.9 |
|  | IMPHY009090 | -8.8 |  | IMPHY002792 | -7.9 |
|  | IMPHY010691 | -8.8 |  | IMPHY002857 | -7.9 |
|  | IMPHY010851 | -8.8 |  | IMPHY002916 | -7.9 |
|  | IMPHY011109 | -8.8 |  | IMPHY002933 | -7.9 |
|  | IMPHY011162 | -8.8 |  | IMPHY002966 | -7.9 |
|  | IMPHY011458 | -8.8 |  | IMPHY002979 | -7.9 |
|  | IMPHY012993 | -8.8 |  | IMPHY003033 | -7.9 |
|  | IMPHY013212 | -8.8 |  | IMPHY003153 | -7.9 |
|  | IMPHY014742 | -8.8 |  | IMPHY003218 | -7.9 |
|  | IMPHY000870 | -8.7 |  | IMPHY003251 | -7.9 |
|  | IMPHY001112 | -8.7 |  | IMPHY003331 | -7.9 |
|  | IMPHY001908 | -8.7 |  | IMPHY003570 | -7.9 |
|  | IMPHY003164 | -8.7 |  | IMPHY003583 | -7.9 |
|  | IMPHY003936 | -8.7 |  | IMPHY003597 | -7.9 |
|  | IMPHY004778 | -8.7 |  | IMPHY003705 | -7.9 |
|  | IMPHY005148 | -8.7 |  | IMPHY003716 | -7.9 |
|  | IMPHY005236 | -8.7 |  | IMPHY003743 | -7.9 |
|  | IMPHY006541 | -8.7 |  | IMPHY003833 | -7.9 |
|  | IMPHY007035 | -8.7 |  | IMPHY003878 | -7.9 |
|  | IMPHY007525 | -8.7 |  | IMPHY003911 | -7.9 |
|  | IMPHY007909 | -8.7 |  | IMPHY003974 | -7.9 |
|  | IMPHY010321 | -8.7 |  | IMPHY004048 | -7.9 |
|  | IMPHY010667 | -8.7 |  | IMPHY004103 | -7.9 |
|  | IMPHY010711 | -8.7 |  | IMPHY004128 | -7.9 |
|  | IMPHY011188 | -8.7 |  | IMPHY004343 | -7.9 |
|  | IMPHY011682 | -8.7 |  | IMPHY004636 | -7.9 |
|  | IMPHY011909 | -8.7 |  | IMPHY004722 | -7.9 |
|  | IMPHY011940 | -8.7 |  | IMPHY004724 | -7.9 |
|  | IMPHY012556 | -8.7 |  | IMPHY004770 | -7.9 |
|  | IMPHY013216 | -8.7 |  | IMPHY004836 | -7.9 |
|  | IMPHY013285 | -8.7 |  | IMPHY005097 | -7.9 |
|  | IMPHY014825 | -8.7 |  | IMPHY005108 | -7.9 |
|  | IMPHY014981 | -8.7 |  | IMPHY005203 | -7.9 |
|  | IMPHY015781 | -8.7 |  | IMPHY005210 | -7.9 |
|  | IMPHY000058 | -8.6 |  | IMPHY005217 | -7.9 |
|  | IMPHY000211 | -8.6 |  | IMPHY005221 | -7.9 |
|  | IMPHY000331 | -8.6 |  | IMPHY005243 | -7.9 |
|  | IMPHY000403 | -8.6 |  | IMPHY005287 | -7.9 |
|  | IMPHY000527 | -8.6 |  | IMPHY005352 | -7.9 |
|  | IMPHY000861 | -8.6 |  | IMPHY005416 | -7.9 |
|  | IMPHY001305 | -8.6 |  | IMPHY005655 | -7.9 |
|  | IMPHY001309 | -8.6 |  | IMPHY005683 | -7.9 |
|  | IMPHY001480 | -8.6 |  | IMPHY005698 | -7.9 |
|  | IMPHY001629 | -8.6 |  | IMPHY005766 | -7.9 |
|  | IMPHY002021 | -8.6 |  | IMPHY005867 | -7.9 |
|  | IMPHY002205 | -8.6 |  | IMPHY005876 | -7.9 |
|  | IMPHY002305 | -8.6 |  | IMPHY006076 | -7.9 |
|  | IMPHY003155 | -8.6 |  | IMPHY006080 | -7.9 |
|  | IMPHY003373 | -8.6 |  | IMPHY006276 | -7.9 |
|  | IMPHY003806 | -8.6 |  | IMPHY006358 | -7.9 |
|  | IMPHY004234 | -8.6 |  | IMPHY006440 | -7.9 |
|  | IMPHY004731 | -8.6 |  | IMPHY006510 | -7.9 |
|  | IMPHY005188 | -8.6 |  | IMPHY006627 | -7.9 |
|  | IMPHY006761 | -8.6 |  | IMPHY006631 | -7.9 |
|  | IMPHY007663 | -8.6 |  | IMPHY006720 | -7.9 |
|  | IMPHY007734 | -8.6 |  | IMPHY006729 | -7.9 |
|  | IMPHY009501 | -8.6 |  | IMPHY006776 | -7.9 |
|  | IMPHY010403 | -8.6 |  | IMPHY006818 | -7.9 |
|  | IMPHY010462 | -8.6 |  | IMPHY006852 | -7.9 |
|  | IMPHY010665 | -8.6 |  | IMPHY006980 | -7.9 |
|  | IMPHY010818 | -8.6 |  | IMPHY007158 | -7.9 |
|  | IMPHY011402 | -8.6 |  | IMPHY007208 | -7.9 |
|  | IMPHY011624 | -8.6 |  | IMPHY007341 | -7.9 |
|  | IMPHY011681 | -8.6 |  | IMPHY007462 | -7.9 |
|  | IMPHY011690 | -8.6 |  | IMPHY007630 | -7.9 |
|  | IMPHY011942 | -8.6 |  | IMPHY007656 | -7.9 |
|  | IMPHY011943 | -8.6 |  | IMPHY007892 | -7.9 |
|  | IMPHY012493 | -8.6 |  | IMPHY007945 | -7.9 |
|  | IMPHY012710 | -8.6 |  | IMPHY007969 | -7.9 |
|  | IMPHY013121 | -8.6 |  | IMPHY008064 | -7.9 |
|  | IMPHY013219 | -8.6 |  | IMPHY008188 | -7.9 |
|  | IMPHY013497 | -8.6 |  | IMPHY008234 | -7.9 |
|  | IMPHY015044 | -8.6 |  | IMPHY008507 | -7.9 |
|  | IMPHY000950 | -8.5 |  | IMPHY008512 | -7.9 |
|  | IMPHY001200 | -8.5 |  | IMPHY008790 | -7.9 |
|  | IMPHY001934 | -8.5 |  | IMPHY009041 | -7.9 |
|  | IMPHY002088 | -8.5 |  | IMPHY009077 | -7.9 |
|  | IMPHY002216 | -8.5 |  | IMPHY009206 | -7.9 |
|  | IMPHY002324 | -8.5 |  | IMPHY009248 | -7.9 |
|  | IMPHY002611 | -8.5 |  | IMPHY009505 | -7.9 |
|  | IMPHY002648 | -8.5 |  | IMPHY009638 | -7.9 |
|  | IMPHY002765 | -8.5 |  | IMPHY009668 | -7.9 |
|  | IMPHY003245 | -8.5 |  | IMPHY009957 | -7.9 |
|  | IMPHY003706 | -8.5 |  | IMPHY010068 | -7.9 |
|  | IMPHY003757 | -8.5 |  | IMPHY010284 | -7.9 |
|  | IMPHY004178 | -8.5 |  | IMPHY010300 | -7.9 |
|  | IMPHY004324 | -8.5 |  | IMPHY010343 | -7.9 |
|  | IMPHY004539 | -8.5 |  | IMPHY010355 | -7.9 |
|  | IMPHY005156 | -8.5 |  | IMPHY010467 | -7.9 |
|  | IMPHY006225 | -8.5 |  | IMPHY010510 | -7.9 |
|  | IMPHY006381 | -8.5 |  | IMPHY010721 | -7.9 |
|  | IMPHY007030 | -8.5 |  | IMPHY010824 | -7.9 |
|  | IMPHY007087 | -8.5 |  | IMPHY010828 | -7.9 |
|  | IMPHY007679 | -8.5 |  | IMPHY010862 | -7.9 |
|  | IMPHY007767 | -8.5 |  | IMPHY010985 | -7.9 |
|  | IMPHY007888 | -8.5 |  | IMPHY011025 | -7.9 |
|  | IMPHY008571 | -8.5 |  | IMPHY011078 | -7.9 |
|  | IMPHY008637 | -8.5 |  | IMPHY011119 | -7.9 |
|  | IMPHY009071 | -8.5 |  | IMPHY011146 | -7.9 |
|  | IMPHY009120 | -8.5 |  | IMPHY011147 | -7.9 |
|  | IMPHY009228 | -8.5 |  | IMPHY011472 | -7.9 |
|  | IMPHY010291 | -8.5 |  | IMPHY011716 | -7.9 |
|  | IMPHY011344 | -8.5 |  | IMPHY011767 | -7.9 |
|  | IMPHY011635 | -8.5 |  | IMPHY011954 | -7.9 |
|  | IMPHY011703 | -8.5 |  | IMPHY011994 | -7.9 |
|  | IMPHY012046 | -8.5 |  | IMPHY012000 | -7.9 |
|  | IMPHY012450 | -8.5 |  | IMPHY012117 | -7.9 |
|  | IMPHY013015 | -8.5 |  | IMPHY012211 | -7.9 |
|  | IMPHY013893 | -8.5 |  | IMPHY012298 | -7.9 |
|  | IMPHY014771 | -8.5 |  | IMPHY012331 | -7.9 |
|  | IMPHY014795 | -8.5 |  | IMPHY012366 | -7.9 |
|  | IMPHY000398 | -8.4 |  | IMPHY012689 | -7.9 |
|  | IMPHY000925 | -8.4 |  | IMPHY012785 | -7.9 |
|  | IMPHY001137 | -8.4 |  | IMPHY012793 | -7.9 |
|  | IMPHY001471 | -8.4 |  | IMPHY012891 | -7.9 |
|  | IMPHY001627 | -8.4 |  | IMPHY012901 | -7.9 |
|  | IMPHY002118 | -8.4 |  | IMPHY013011 | -7.9 |
|  | IMPHY002349 | -8.4 |  | IMPHY013069 | -7.9 |
|  | IMPHY002579 | -8.4 |  | IMPHY013082 | -7.9 |
|  | IMPHY002619 | -8.4 |  | IMPHY013180 | -7.9 |
|  | IMPHY002660 | -8.4 |  | IMPHY013192 | -7.9 |
|  | IMPHY002700 | -8.4 |  | IMPHY013430 | -7.9 |
|  | IMPHY003320 | -8.4 |  | IMPHY013437 | -7.9 |
|  | IMPHY004278 | -8.4 |  | IMPHY013522 | -7.9 |
|  | IMPHY004284 | -8.4 |  | IMPHY013554 | -7.9 |
|  | IMPHY004719 | -8.4 |  | IMPHY013580 | -7.9 |
|  | IMPHY005049 | -8.4 |  | IMPHY013593 | -7.9 |
|  | IMPHY005195 | -8.4 |  | IMPHY013921 | -7.9 |
|  | IMPHY005227 | -8.4 |  | IMPHY014158 | -7.9 |
|  | IMPHY005568 | -8.4 |  | IMPHY014243 | -7.9 |
|  | IMPHY006067 | -8.4 |  | IMPHY014251 | -7.9 |
|  | IMPHY006587 | -8.4 |  | IMPHY014321 | -7.9 |
|  | IMPHY007015 | -8.4 |  | IMPHY014481 | -7.9 |
|  | IMPHY007742 | -8.4 |  | IMPHY014738 | -7.9 |
|  | IMPHY008157 | -8.4 |  | IMPHY015068 | -7.9 |
|  | IMPHY008174 | -8.4 |  | IMPHY015090 | -7.9 |
|  | IMPHY008900 | -8.4 |  | IMPHY015486 | -7.9 |
|  | IMPHY009050 | -8.4 |  | IMPHY015880 | -7.9 |
|  | IMPHY009916 | -8.4 |  | IMPHY016991 | -7.9 |
|  | IMPHY010367 | -8.4 |  | IMPHY000049 | -7.8 |
|  | IMPHY010419 | -8.4 |  | IMPHY000068 | -7.8 |
|  | IMPHY010999 | -8.4 |  | IMPHY000081 | -7.8 |
|  | IMPHY011376 | -8.4 |  | IMPHY000220 | -7.8 |
|  | IMPHY011420 | -8.4 |  | IMPHY000233 | -7.8 |
|  | IMPHY011701 | -8.4 |  | IMPHY000414 | -7.8 |
|  | IMPHY012846 | -8.4 |  | IMPHY000449 | -7.8 |
|  | IMPHY013041 | -8.4 |  | IMPHY000596 | -7.8 |
|  | IMPHY013253 | -8.4 |  | IMPHY000611 | -7.8 |
|  | IMPHY013311 | -8.4 |  | IMPHY000615 | -7.8 |
|  | IMPHY013362 | -8.4 |  | IMPHY000655 | -7.8 |
|  | IMPHY013429 | -8.4 |  | IMPHY000712 | -7.8 |
|  | IMPHY013601 | -8.4 |  | IMPHY000819 | -7.8 |
|  | IMPHY013913 | -8.4 |  | IMPHY001157 | -7.8 |
|  | IMPHY013962 | -8.4 |  | IMPHY001202 | -7.8 |
|  | IMPHY014201 | -8.4 |  | IMPHY001243 | -7.8 |
|  | IMPHY014598 | -8.4 |  | IMPHY001295 | -7.8 |
|  | IMPHY014974 | -8.4 |  | IMPHY001463 | -7.8 |
|  | IMPHY015110 | -8.4 |  | IMPHY001568 | -7.8 |
|  | IMPHY000159 | -8.3 |  | IMPHY001607 | -7.8 |
|  | IMPHY000296 | -8.3 |  | IMPHY001612 | -7.8 |
|  | IMPHY000576 | -8.3 |  | IMPHY001887 | -7.8 |
|  | IMPHY000643 | -8.3 |  | IMPHY001889 | -7.8 |
|  | IMPHY000860 | -8.3 |  | IMPHY001898 | -7.8 |
|  | IMPHY000890 | -8.3 |  | IMPHY001973 | -7.8 |
|  | IMPHY001769 | -8.3 |  | IMPHY001998 | -7.8 |
|  | IMPHY001782 | -8.3 |  | IMPHY002119 | -7.8 |
|  | IMPHY002001 | -8.3 |  | IMPHY002144 | -7.8 |
|  | IMPHY002077 | -8.3 |  | IMPHY002188 | -7.8 |
|  | IMPHY002117 | -8.3 |  | IMPHY002449 | -7.8 |
|  | IMPHY002638 | -8.3 |  | IMPHY002547 | -7.8 |
|  | IMPHY002653 | -8.3 |  | IMPHY002600 | -7.8 |
|  | IMPHY002757 | -8.3 |  | IMPHY002747 | -7.8 |
|  | IMPHY003272 | -8.3 |  | IMPHY002776 | -7.8 |
|  | IMPHY003364 | -8.3 |  | IMPHY003292 | -7.8 |
|  | IMPHY003407 | -8.3 |  | IMPHY003329 | -7.8 |
|  | IMPHY003542 | -8.3 |  | IMPHY003433 | -7.8 |
|  | IMPHY003572 | -8.3 |  | IMPHY003487 | -7.8 |
|  | IMPHY003808 | -8.3 |  | IMPHY003683 | -7.8 |
|  | IMPHY003859 | -8.3 |  | IMPHY003758 | -7.8 |
|  | IMPHY003860 | -8.3 |  | IMPHY003771 | -7.8 |
|  | IMPHY003991 | -8.3 |  | IMPHY004243 | -7.8 |
|  | IMPHY004277 | -8.3 |  | IMPHY004244 | -7.8 |
|  | IMPHY004465 | -8.3 |  | IMPHY004491 | -7.8 |
|  | IMPHY005362 | -8.3 |  | IMPHY004758 | -7.8 |
|  | IMPHY005446 | -8.3 |  | IMPHY004759 | -7.8 |
|  | IMPHY005738 | -8.3 |  | IMPHY004858 | -7.8 |
|  | IMPHY005751 | -8.3 |  | IMPHY004931 | -7.8 |
|  | IMPHY006119 | -8.3 |  | IMPHY004935 | -7.8 |
|  | IMPHY007333 | -8.3 |  | IMPHY004957 | -7.8 |
|  | IMPHY007463 | -8.3 |  | IMPHY004974 | -7.8 |
|  | IMPHY007501 | -8.3 |  | IMPHY005110 | -7.8 |
|  | IMPHY007652 | -8.3 |  | IMPHY005114 | -7.8 |
|  | IMPHY008010 | -8.3 |  | IMPHY005200 | -7.8 |
|  | IMPHY008202 | -8.3 |  | IMPHY005246 | -7.8 |
|  | IMPHY008334 | -8.3 |  | IMPHY005385 | -7.8 |
|  | IMPHY008417 | -8.3 |  | IMPHY005493 | -7.8 |
|  | IMPHY008618 | -8.3 |  | IMPHY005515 | -7.8 |
|  | IMPHY008826 | -8.3 |  | IMPHY005535 | -7.8 |
|  | IMPHY008964 | -8.3 |  | IMPHY005633 | -7.8 |
|  | IMPHY009188 | -8.3 |  | IMPHY005892 | -7.8 |
|  | IMPHY009189 | -8.3 |  | IMPHY006090 | -7.8 |
|  | IMPHY009234 | -8.3 |  | IMPHY006097 | -7.8 |
|  | IMPHY010295 | -8.3 |  | IMPHY006303 | -7.8 |
|  | IMPHY010366 | -8.3 |  | IMPHY006443 | -7.8 |
|  | IMPHY010369 | -8.3 |  | IMPHY006462 | -7.8 |
|  | IMPHY010516 | -8.3 |  | IMPHY006480 | -7.8 |
|  | IMPHY010572 | -8.3 |  | IMPHY006483 | -7.8 |
|  | IMPHY010919 | -8.3 |  | IMPHY006501 | -7.8 |
|  | IMPHY011452 | -8.3 |  | IMPHY006586 | -7.8 |
|  | IMPHY011634 | -8.3 |  | IMPHY006624 | -7.8 |
|  | IMPHY011952 | -8.3 |  | IMPHY006634 | -7.8 |
|  | IMPHY011993 | -8.3 |  | IMPHY006673 | -7.8 |
|  | IMPHY012387 | -8.3 |  | IMPHY006697 | -7.8 |
|  | IMPHY012578 | -8.3 |  | IMPHY006727 | -7.8 |
|  | IMPHY012878 | -8.3 |  | IMPHY006756 | -7.8 |
|  | IMPHY012968 | -8.3 |  | IMPHY006771 | -7.8 |
|  | IMPHY013142 | -8.3 |  | IMPHY006808 | -7.8 |
|  | IMPHY013220 | -8.3 |  | IMPHY006880 | -7.8 |
|  | IMPHY013396 | -8.3 |  | IMPHY006919 | -7.8 |
|  | IMPHY014011 | -8.3 |  | IMPHY006927 | -7.8 |
|  | IMPHY014059 | -8.3 |  | IMPHY006973 | -7.8 |
|  | IMPHY014571 | -8.3 |  | IMPHY006974 | -7.8 |
|  | IMPHY014613 | -8.3 |  | IMPHY007111 | -7.8 |
|  | IMPHY014725 | -8.3 |  | IMPHY007332 | -7.8 |
|  | IMPHY014736 | -8.3 |  | IMPHY007572 | -7.8 |
|  | IMPHY014859 | -8.3 |  | IMPHY007597 | -7.8 |
|  | IMPHY000090 | -8.2 |  | IMPHY007854 | -7.8 |
|  | IMPHY000565 | -8.2 |  | IMPHY007899 | -7.8 |
|  | IMPHY000582 | -8.2 |  | IMPHY007966 | -7.8 |
|  | IMPHY000677 | -8.2 |  | IMPHY008072 | -7.8 |
|  | IMPHY000710 | -8.2 |  | IMPHY008081 | -7.8 |
|  | IMPHY001197 | -8.2 |  | IMPHY008117 | -7.8 |
|  | IMPHY001229 | -8.2 |  | IMPHY008138 | -7.8 |
|  | IMPHY001232 | -8.2 |  | IMPHY008289 | -7.8 |
|  | IMPHY001469 | -8.2 |  | IMPHY008291 | -7.8 |
|  | IMPHY001637 | -8.2 |  | IMPHY008413 | -7.8 |
|  | IMPHY001660 | -8.2 |  | IMPHY008460 | -7.8 |
|  | IMPHY001678 | -8.2 |  | IMPHY008476 | -7.8 |
|  | IMPHY001883 | -8.2 |  | IMPHY008490 | -7.8 |
|  | IMPHY002057 | -8.2 |  | IMPHY008573 | -7.8 |
|  | IMPHY002068 | -8.2 |  | IMPHY008617 | -7.8 |
|  | IMPHY002146 | -8.2 |  | IMPHY008668 | -7.8 |
|  | IMPHY002279 | -8.2 |  | IMPHY008831 | -7.8 |
|  | IMPHY002408 | -8.2 |  | IMPHY008838 | -7.8 |
|  | IMPHY002621 | -8.2 |  | IMPHY008870 | -7.8 |
|  | IMPHY002828 | -8.2 |  | IMPHY008966 | -7.8 |
|  | IMPHY003066 | -8.2 |  | IMPHY009059 | -7.8 |
|  | IMPHY003367 | -8.2 |  | IMPHY009119 | -7.8 |
|  | IMPHY003612 | -8.2 |  | IMPHY009347 | -7.8 |
|  | IMPHY003772 | -8.2 |  | IMPHY009380 | -7.8 |
|  | IMPHY003954 | -8.2 |  | IMPHY009427 | -7.8 |
|  | IMPHY004377 | -8.2 |  | IMPHY009464 | -7.8 |
|  | IMPHY004384 | -8.2 |  | IMPHY009493 | -7.8 |
|  | IMPHY004577 | -8.2 |  | IMPHY009584 | -7.8 |
|  | IMPHY004579 | -8.2 |  | IMPHY009586 | -7.8 |
|  | IMPHY004823 | -8.2 |  | IMPHY009647 | -7.8 |
|  | IMPHY004825 | -8.2 |  | IMPHY009649 | -7.8 |
|  | IMPHY005166 | -8.2 |  | IMPHY009793 | -7.8 |
|  | IMPHY005229 | -8.2 |  | IMPHY009913 | -7.8 |
|  | IMPHY005278 | -8.2 |  | IMPHY010313 | -7.8 |
|  | IMPHY005339 | -8.2 |  | IMPHY010383 | -7.8 |
|  | IMPHY005703 | -8.2 |  | IMPHY010474 | -7.8 |
|  | IMPHY005877 | -8.2 |  | IMPHY010702 | -7.8 |
|  | IMPHY005903 | -8.2 |  | IMPHY010714 | -7.8 |
|  | IMPHY006167 | -8.2 |  | IMPHY011028 | -7.8 |
|  | IMPHY006363 | -8.2 |  | IMPHY011132 | -7.8 |
|  | IMPHY006549 | -8.2 |  | IMPHY011160 | -7.8 |
|  | IMPHY006703 | -8.2 |  | IMPHY011204 | -7.8 |
|  | IMPHY007164 | -8.2 |  | IMPHY011238 | -7.8 |
|  | IMPHY007371 | -8.2 |  | IMPHY011257 | -7.8 |
|  | IMPHY007613 | -8.2 |  | IMPHY011268 | -7.8 |
|  | IMPHY007729 | -8.2 |  | IMPHY011318 | -7.8 |
|  | IMPHY007891 | -8.2 |  | IMPHY011364 | -7.8 |
|  | IMPHY007947 | -8.2 |  | IMPHY011442 | -7.8 |
|  | IMPHY007952 | -8.2 |  | IMPHY011543 | -7.8 |
|  | IMPHY008136 | -8.2 |  | IMPHY011664 | -7.8 |
|  | IMPHY008346 | -8.2 |  | IMPHY011704 | -7.8 |
|  | IMPHY008404 | -8.2 |  | IMPHY011765 | -7.8 |
|  | IMPHY008427 | -8.2 |  | IMPHY011847 | -7.8 |
|  | IMPHY008566 | -8.2 |  | IMPHY011948 | -7.8 |
|  | IMPHY008579 | -8.2 |  | IMPHY012210 | -7.8 |
|  | IMPHY008586 | -8.2 |  | IMPHY012299 | -7.8 |
|  | IMPHY008651 | -8.2 |  | IMPHY012358 | -7.8 |
|  | IMPHY008760 | -8.2 |  | IMPHY012435 | -7.8 |
|  | IMPHY008794 | -8.2 |  | IMPHY012646 | -7.8 |
|  | IMPHY008874 | -8.2 |  | IMPHY012833 | -7.8 |
|  | IMPHY009023 | -8.2 |  | IMPHY012838 | -7.8 |
|  | IMPHY009031 | -8.2 |  | IMPHY012850 | -7.8 |
|  | IMPHY009038 | -8.2 |  | IMPHY012876 | -7.8 |
|  | IMPHY009083 | -8.2 |  | IMPHY012890 | -7.8 |
|  | IMPHY009269 | -8.2 |  | IMPHY012992 | -7.8 |
|  | IMPHY010024 | -8.2 |  | IMPHY013046 | -7.8 |
|  | IMPHY010181 | -8.2 |  | IMPHY013057 | -7.8 |
|  | IMPHY010194 | -8.2 |  | IMPHY013106 | -7.8 |
|  | IMPHY010228 | -8.2 |  | IMPHY013189 | -7.8 |
|  | IMPHY010341 | -8.2 |  | IMPHY013194 | -7.8 |
|  | IMPHY010395 | -8.2 |  | IMPHY013255 | -7.8 |
|  | IMPHY010399 | -8.2 |  | IMPHY013264 | -7.8 |
|  | IMPHY010431 | -8.2 |  | IMPHY013269 | -7.8 |
|  | IMPHY010638 | -8.2 |  | IMPHY013360 | -7.8 |
|  | IMPHY010705 | -8.2 |  | IMPHY013415 | -7.8 |
|  | IMPHY010858 | -8.2 |  | IMPHY013427 | -7.8 |
|  | IMPHY011246 | -8.2 |  | IMPHY013466 | -7.8 |
|  | IMPHY011665 | -8.2 |  | IMPHY013565 | -7.8 |
|  | IMPHY011981 | -8.2 |  | IMPHY013616 | -7.8 |
|  | IMPHY012460 | -8.2 |  | IMPHY014004 | -7.8 |
|  | IMPHY012495 | -8.2 |  | IMPHY014015 | -7.8 |
|  | IMPHY012869 | -8.2 |  | IMPHY014143 | -7.8 |
|  | IMPHY012899 | -8.2 |  | IMPHY014258 | -7.8 |
|  | IMPHY013006 | -8.2 |  | IMPHY014326 | -7.8 |
|  | IMPHY013435 | -8.2 |  | IMPHY014336 | -7.8 |
|  | IMPHY014335 | -8.2 |  | IMPHY014399 | -7.8 |
|  | IMPHY014375 | -8.2 |  | IMPHY014442 | -7.8 |
|  | IMPHY014529 | -8.2 |  | IMPHY014509 | -7.8 |
|  | IMPHY014734 | -8.2 |  | IMPHY014677 | -7.8 |
|  | IMPHY015018 | -8.2 |  | IMPHY014730 | -7.8 |
|  | IMPHY015070 | -8.2 |  | IMPHY014939 | -7.8 |
|  | IMPHY000186 | -8.1 |  | IMPHY016032 | -7.8 |
|  | IMPHY000196 | -8.1 |  | IMPHY016905 | -7.8 |
|  | IMPHY000342 | -8.1 |  | IMPHY017818 | -7.8 |
|  | IMPHY000354 | -8.1 |  | IMPHY000097 | -7.7 |
|  | IMPHY000478 | -8.1 |  | IMPHY000104 | -7.7 |
|  | IMPHY000529 | -8.1 |  | IMPHY000151 | -7.7 |
|  | IMPHY000840 | -8.1 |  | IMPHY000166 | -7.7 |
|  | IMPHY000852 | -8.1 |  | IMPHY000304 | -7.7 |
|  | IMPHY001254 | -8.1 |  | IMPHY000390 | -7.7 |
|  | IMPHY001276 | -8.1 |  | IMPHY000802 | -7.7 |
|  | IMPHY001417 | -8.1 |  | IMPHY000964 | -7.7 |
|  | IMPHY001462 | -8.1 |  | IMPHY000969 | -7.7 |
|  | IMPHY001530 | -8.1 |  | IMPHY001052 | -7.7 |
|  | IMPHY001734 | -8.1 |  | IMPHY001055 | -7.7 |
|  | IMPHY001798 | -8.1 |  | IMPHY001091 | -7.7 |
|  | IMPHY001948 | -8.1 |  | IMPHY001124 | -7.7 |
|  | IMPHY002152 | -8.1 |  | IMPHY001147 | -7.7 |
|  | IMPHY002235 | -8.1 |  | IMPHY001169 | -7.7 |
|  | IMPHY002300 | -8.1 |  | IMPHY001206 | -7.7 |
|  | IMPHY002335 | -8.1 |  | IMPHY001349 | -7.7 |
|  | IMPHY002338 | -8.1 |  | IMPHY001404 | -7.7 |
|  | IMPHY002354 | -8.1 |  | IMPHY001408 | -7.7 |
|  | IMPHY002447 | -8.1 |  | IMPHY001451 | -7.7 |
|  | IMPHY002522 | -8.1 |  | IMPHY001455 | -7.7 |
|  | IMPHY002530 | -8.1 |  | IMPHY001464 | -7.7 |
|  | IMPHY002540 | -8.1 |  | IMPHY001485 | -7.7 |
|  | IMPHY002578 | -8.1 |  | IMPHY001506 | -7.7 |
|  | IMPHY002669 | -8.1 |  | IMPHY001561 | -7.7 |
|  | IMPHY002777 | -8.1 |  | IMPHY001601 | -7.7 |
|  | IMPHY002805 | -8.1 |  | IMPHY001677 | -7.7 |
|  | IMPHY002990 | -8.1 |  | IMPHY001791 | -7.7 |
|  | IMPHY003379 | -8.1 |  | IMPHY001904 | -7.7 |
|  | IMPHY003509 | -8.1 |  | IMPHY002096 | -7.7 |
|  | IMPHY003595 | -8.1 |  | IMPHY002157 | -7.7 |
|  | IMPHY003644 | -8.1 |  | IMPHY002224 | -7.7 |
|  | IMPHY003698 | -8.1 |  | IMPHY002285 | -7.7 |
|  | IMPHY004132 | -8.1 |  | IMPHY002348 | -7.7 |
|  | IMPHY004177 | -8.1 |  | IMPHY002414 | -7.7 |
|  | IMPHY004487 | -8.1 |  | IMPHY002420 | -7.7 |
|  | IMPHY004720 | -8.1 |  | IMPHY002470 | -7.7 |
|  | IMPHY004802 | -8.1 |  | IMPHY002503 | -7.7 |
|  | IMPHY004835 | -8.1 |  | IMPHY002585 | -7.7 |
|  | IMPHY004897 | -8.1 |  | IMPHY002696 | -7.7 |
|  | IMPHY004937 | -8.1 |  | IMPHY002811 | -7.7 |
|  | IMPHY004959 | -8.1 |  | IMPHY002813 | -7.7 |
|  | IMPHY005005 | -8.1 |  | IMPHY002816 | -7.7 |
|  | IMPHY005151 | -8.1 |  | IMPHY002840 | -7.7 |
|  | IMPHY005179 | -8.1 |  | IMPHY002868 | -7.7 |
|  | IMPHY005448 | -8.1 |  | IMPHY002902 | -7.7 |
|  | IMPHY005482 | -8.1 |  | IMPHY002922 | -7.7 |
|  | IMPHY005520 | -8.1 |  | IMPHY003085 | -7.7 |
|  | IMPHY005537 | -8.1 |  | IMPHY003184 | -7.7 |
|  | IMPHY005888 | -8.1 |  | IMPHY003223 | -7.7 |
|  | IMPHY006271 | -8.1 |  | IMPHY003234 | -7.7 |
|  | IMPHY006294 | -8.1 |  | IMPHY003273 | -7.7 |
|  | IMPHY006313 | -8.1 |  | IMPHY003321 | -7.7 |
|  | IMPHY006665 | -8.1 |  | IMPHY003354 | -7.7 |
|  | IMPHY006688 | -8.1 |  | IMPHY003527 | -7.7 |
|  | IMPHY006730 | -8.1 |  | IMPHY003569 | -7.7 |
|  | IMPHY006766 | -8.1 |  | IMPHY003646 | -7.7 |
|  | IMPHY006854 | -8.1 |  | IMPHY003700 | -7.7 |
|  | IMPHY007662 | -8.1 |  | IMPHY003805 | -7.7 |
|  | IMPHY007770 | -8.1 |  | IMPHY003823 | -7.7 |
|  | IMPHY007900 | -8.1 |  | IMPHY004255 | -7.7 |
|  | IMPHY007935 | -8.1 |  | IMPHY004340 | -7.7 |
|  | IMPHY008016 | -8.1 |  | IMPHY004357 | -7.7 |
|  | IMPHY008113 | -8.1 |  | IMPHY004412 | -7.7 |
|  | IMPHY008185 | -8.1 |  | IMPHY004416 | -7.7 |
|  | IMPHY008389 | -8.1 |  | IMPHY004497 | -7.7 |
|  | IMPHY008462 | -8.1 |  | IMPHY004714 | -7.7 |
|  | IMPHY008594 | -8.1 |  | IMPHY004740 | -7.7 |
|  | IMPHY008887 | -8.1 |  | IMPHY004754 | -7.7 |
|  | IMPHY008961 | -8.1 |  | IMPHY004763 | -7.7 |
|  | IMPHY009064 | -8.1 |  | IMPHY004773 | -7.7 |
|  | IMPHY009174 | -8.1 |  | IMPHY004781 | -7.7 |
|  | IMPHY009275 | -8.1 |  | IMPHY004844 | -7.7 |
|  | IMPHY009324 | -8.1 |  | IMPHY004879 | -7.7 |
|  | IMPHY009331 | -8.1 |  | IMPHY005055 | -7.7 |
|  | IMPHY009386 | -8.1 |  | IMPHY005059 | -7.7 |
|  | IMPHY009518 | -8.1 |  | IMPHY005214 | -7.7 |
|  | IMPHY010169 | -8.1 |  | IMPHY005503 | -7.7 |
|  | IMPHY010197 | -8.1 |  | IMPHY005739 | -7.7 |
|  | IMPHY010730 | -8.1 |  | IMPHY005860 | -7.7 |
|  | IMPHY010821 | -8.1 |  | IMPHY005909 | -7.7 |
|  | IMPHY011259 | -8.1 |  | IMPHY005914 | -7.7 |
|  | IMPHY011547 | -8.1 |  | IMPHY006051 | -7.7 |
|  | IMPHY011650 | -8.1 |  | IMPHY006072 | -7.7 |
|  | IMPHY011706 | -8.1 |  | IMPHY006158 | -7.7 |
|  | IMPHY011813 | -8.1 |  | IMPHY006161 | -7.7 |
|  | IMPHY011908 | -8.1 |  | IMPHY006210 | -7.7 |
|  | IMPHY011955 | -8.1 |  | IMPHY006630 | -7.7 |
|  | IMPHY012317 | -8.1 |  | IMPHY006712 | -7.7 |
|  | IMPHY012443 | -8.1 |  | IMPHY006738 | -7.7 |
|  | IMPHY012446 | -8.1 |  | IMPHY006874 | -7.7 |
|  | IMPHY012506 | -8.1 |  | IMPHY007002 | -7.7 |
|  | IMPHY012553 | -8.1 |  | IMPHY007144 | -7.7 |
|  | IMPHY012669 | -8.1 |  | IMPHY007255 | -7.7 |
|  | IMPHY012674 | -8.1 |  | IMPHY007382 | -7.7 |
|  | IMPHY012730 | -8.1 |  | IMPHY007502 | -7.7 |
|  | IMPHY012783 | -8.1 |  | IMPHY007551 | -7.7 |
|  | IMPHY012987 | -8.1 |  | IMPHY007552 | -7.7 |
|  | IMPHY013182 | -8.1 |  | IMPHY007621 | -7.7 |
|  | IMPHY013227 | -8.1 |  | IMPHY007694 | -7.7 |
|  | IMPHY013258 | -8.1 |  | IMPHY007697 | -7.7 |
|  | IMPHY013305 | -8.1 |  | IMPHY007793 | -7.7 |
|  | IMPHY013361 | -8.1 |  | IMPHY007879 | -7.7 |
|  | IMPHY013946 | -8.1 |  | IMPHY008026 | -7.7 |
|  | IMPHY014176 | -8.1 |  | IMPHY008032 | -7.7 |
|  | IMPHY015024 | -8.1 |  | IMPHY008033 | -7.7 |
|  | IMPHY015025 | -8.1 |  | IMPHY008160 | -7.7 |
|  | IMPHY015779 | -8.1 |  | IMPHY008167 | -7.7 |
|  | IMPHY015793 | -8.1 |  | IMPHY008283 | -7.7 |
|  | IMPHY016842 | -8.1 |  | IMPHY008409 | -7.7 |
|  | IMPHY000086 | -8 |  | IMPHY008435 | -7.7 |
|  | IMPHY000322 | -8 |  | IMPHY008449 | -7.7 |
|  | IMPHY000330 | -8 |  | IMPHY008562 | -7.7 |
|  | IMPHY000346 | -8 |  | IMPHY008600 | -7.7 |
|  | IMPHY000353 | -8 |  | IMPHY008610 | -7.7 |
|  | IMPHY000375 | -8 |  | IMPHY008735 | -7.7 |
|  | IMPHY000446 | -8 |  | IMPHY008782 | -7.7 |
|  | IMPHY000534 | -8 |  | IMPHY009098 | -7.7 |
|  | IMPHY000727 | -8 |  | IMPHY009243 | -7.7 |
|  | IMPHY000863 | -8 |  | IMPHY009391 | -7.7 |
|  | IMPHY000982 | -8 |  | IMPHY009424 | -7.7 |
|  | IMPHY001088 | -8 |  | IMPHY009548 | -7.7 |
|  | IMPHY001695 | -8 |  | IMPHY010079 | -7.7 |
|  | IMPHY001770 | -8 |  | IMPHY010150 | -7.7 |
|  | IMPHY001790 | -8 |  | IMPHY010173 | -7.7 |
|  | IMPHY001907 | -8 |  | IMPHY010193 | -7.7 |
|  | IMPHY002058 | -8 |  | IMPHY010279 | -7.7 |
|  | IMPHY002436 | -8 |  | IMPHY010379 | -7.7 |
|  | IMPHY002476 | -8 |  | IMPHY010427 | -7.7 |
|  | IMPHY002481 | -8 |  | IMPHY010454 | -7.7 |
|  | IMPHY002644 | -8 |  | IMPHY010539 | -7.7 |
|  | IMPHY002674 | -8 |  | IMPHY010555 | -7.7 |
|  | IMPHY002753 | -8 |  | IMPHY010589 | -7.7 |
|  | IMPHY002793 | -8 |  | IMPHY010672 | -7.7 |
|  | IMPHY002806 | -8 |  | IMPHY010707 | -7.7 |
|  | IMPHY003135 | -8 |  | IMPHY010769 | -7.7 |
|  | IMPHY003213 | -8 |  | IMPHY010804 | -7.7 |
|  | IMPHY003652 | -8 |  | IMPHY010863 | -7.7 |
|  | IMPHY003663 | -8 |  | IMPHY010936 | -7.7 |
|  | IMPHY004118 | -8 |  | IMPHY011117 | -7.7 |
|  | IMPHY004346 | -8 |  | IMPHY011424 | -7.7 |
|  | IMPHY004395 | -8 |  | IMPHY011566 | -7.7 |
|  | IMPHY004472 | -8 |  | IMPHY011610 | -7.7 |
|  | IMPHY004592 | -8 |  | IMPHY011702 | -7.7 |
|  | IMPHY004707 | -8 |  | IMPHY012006 | -7.7 |
|  | IMPHY004721 | -8 |  | IMPHY012270 | -7.7 |
|  | IMPHY004783 | -8 |  | IMPHY012282 | -7.7 |
|  | IMPHY004803 | -8 |  | IMPHY012335 | -7.7 |
|  | IMPHY004824 | -8 |  | IMPHY012338 | -7.7 |
|  | IMPHY004899 | -8 |  | IMPHY012353 | -7.7 |
|  | IMPHY004927 | -8 |  | IMPHY012542 | -7.7 |
|  | IMPHY005095 | -8 |  | IMPHY012688 | -7.7 |
|  | IMPHY005125 | -8 |  | IMPHY012695 | -7.7 |
|  | IMPHY005177 | -8 |  | IMPHY012924 | -7.7 |
|  | IMPHY005185 | -8 |  | IMPHY013074 | -7.7 |
|  | IMPHY005187 | -8 |  | IMPHY013226 | -7.7 |
|  | IMPHY005247 | -8 |  | IMPHY013251 | -7.7 |
|  | IMPHY005274 | -8 |  | IMPHY013369 | -7.7 |
|  | IMPHY005282 | -8 |  | IMPHY013371 | -7.7 |
|  | IMPHY005310 | -8 |  | IMPHY013394 | -7.7 |
|  | IMPHY005361 | -8 |  | IMPHY013399 | -7.7 |
|  | IMPHY005478 | -8 |  | IMPHY013926 | -7.7 |
|  | IMPHY005565 | -8 |  | IMPHY013931 | -7.7 |
|  | IMPHY005665 | -8 |  | IMPHY014037 | -7.7 |
|  | IMPHY005701 | -8 |  | IMPHY014108 | -7.7 |
|  | IMPHY005916 | -8 |  | IMPHY014168 | -7.7 |
|  | IMPHY006042 | -8 |  | IMPHY014226 | -7.7 |
|  | IMPHY006135 | -8 |  | IMPHY014312 | -7.7 |
|  | IMPHY006364 | -8 |  | IMPHY014523 | -7.7 |
|  | IMPHY006413 | -8 |  | IMPHY014537 | -7.7 |
|  | IMPHY006603 | -8 |  | IMPHY014544 | -7.7 |
|  | IMPHY006701 | -8 |  | IMPHY014585 | -7.7 |
|  | IMPHY006786 | -8 |  | IMPHY014848 | -7.7 |
|  | IMPHY006798 | -8 |  | IMPHY015707 | -7.7 |
|  | IMPHY006893 | -8 |  | IMPHY015794 | -7.7 |
|  | IMPHY007336 | -8 |  | IMPHY001442 | -7.6 |
|  | IMPHY007408 | -8 |  | IMPHY002686 | -7.6 |
|  | IMPHY007461 | -8 |  | IMPHY002848 | -7.6 |
|  | IMPHY007489 | -8 |  | IMPHY003011 | -7.6 |
|  | IMPHY007495 | -8 |  | IMPHY003058 | -7.6 |
|  | IMPHY007667 | -8 |  | IMPHY003064 | -7.6 |
|  | IMPHY007730 | -8 |  | IMPHY003770 | -7.6 |
|  | IMPHY007918 | -8 |  | IMPHY004920 | -7.6 |
|  | IMPHY008058 | -8 |  | IMPHY005224 | -7.6 |
|  | IMPHY008403 | -8 |  | IMPHY006918 | -7.6 |
|  | IMPHY008442 | -8 |  | IMPHY007028 | -7.6 |
|  | IMPHY008466 | -8 |  | IMPHY007801 | -7.6 |
|  | IMPHY008499 | -8 |  | IMPHY009204 | -7.6 |
|  | IMPHY008565 | -8 |  | IMPHY012406 | -7.6 |
|  | IMPHY008608 | -8 |  | IMPHY014167 | -7.6 |
|  | IMPHY008619 | -8 |  | IMPHY013211 | -7.5 |
|  | IMPHY009010 | -8 |  | IMPHY001527 | -7.4 |
|  | IMPHY009049 | -8 |  | IMPHY003094 | -7.4 |
|  | IMPHY009060 | -8 |  | IMPHY004447 | -7.4 |
|  | IMPHY009092 | -8 |  | IMPHY008537 | -7.4 |
