# Supplementary material for: Exploring phytochemicals as potential pharmacological inhibitors for NS1 protein of Kyasanur forest disease virus using virtual screening, molecular docking, and molecular simulation approach
Source: PeerJ. 2025 Oct 9;13:e19954. doi: 10.7717/peerj.19954 (PMC12515432; doi:10.7717/peerj.19954)
Supplement: Supplemental Information 3 [file peerj-13-19954-s003.docx]

**Table S3**: Tertiary Virtual screening of 1150 compounds with binding energy value by AutoDock 4.0 implemented in PyRx software

| **Sr.No.** | **Ligand Name** | **Binding Energy (kcal/mol)** | **Sr.No.** | **Ligand Name** | **Binding Energy**  **(kcal/mol)** |
| --- | --- | --- | --- | --- | --- |
|  | IMPHY000366 | -9.76 |  | IMPHY009067 | -7.68 |
|  | IMPHY010294 | -9.34 |  | IMPHY003373 | -7.68 |
|  | IMPHY001281 | -9.12 |  | IMPHY004515 | -7.67 |
|  | IMPHY004082 | -9.08 |  | IMPHY007957 | -7.66 |
|  | IMPHY011162 | -9.08 |  | IMPHY002121 | -7.63 |
|  | IMPHY003352 | -8.99 |  | IMPHY010851 | -7.62 |
|  | IMPHY001309 | -8.94 |  | IMPHY005667 | -7.59 |
|  | IMPHY010476 | -8.93 |  | IMPHY001112 | -7.59 |
|  | IMPHY001575 | -8.92 |  | IMPHY011682 | -7.57 |
|  | IMPHY008834 | -8.92 |  | IMPHY002021 | -7.55 |
|  | IMPHY001343 | -8.85 |  | IMPHY005236 | -7.54 |
|  | IMPHY009308 | -8.83 |  | IMPHY013285 | -7.5 |
|  | IMPHY008030 | -8.83 |  | IMPHY002382 | -7.49 |
|  | IMPHY004035 | -8.7 |  | IMPHY012993 | -7.43 |
|  | IMPHY004234 | -8.7 |  | IMPHY003078 | -7.42 |
|  | IMPHY009264 | -8.68 |  | IMPHY014170 | -7.42 |
|  | IMPHY005266 | -8.66 |  | IMPHY011909 | -7.38 |
|  | IMPHY011945 | -8.64 |  | IMPHY017577 | -7.35 |
|  | IMPHY014742 | -8.63 |  | IMPHY000058 | -7.34 |
|  | IMPHY004140 | -8.62 |  | IMPHY013216 | -7.31 |
|  | IMPHY000317 | -8.57 |  | IMPHY000861 | -7.28 |
|  | IMPHY010666 | -8.55 |  | IMPHY011188 | -7.26 |
|  | IMPHY002305 | -8.44 |  | IMPHY010691 | -7.24 |
|  | IMPHY006541 | -8.41 |  | IMPHY003143 | -7.23 |
|  | IMPHY012275 | -8.38 |  | IMPHY004968 | -7.17 |
|  | IMPHY004849 | -8.37 |  | IMPHY003529 | -7.17 |
|  | IMPHY005293 | -8.35 |  | IMPHY004778 | -7.16 |
|  | IMPHY005775 | -8.34 |  | IMPHY002205 | -7.16 |
|  | IMPHY004287 | -8.34 |  | IMPHY001305 | -7.13 |
|  | IMPHY010667 | -8.34 |  | IMPHY004731 | -7.13 |
|  | IMPHY014146 | -8.32 |  | IMPHY015781 | -7.11 |
|  | IMPHY005148 | -8.32 |  | IMPHY009244 | -7.09 |
|  | IMPHY014825 | -8.3 |  | IMPHY006318 | -7.08 |
|  | IMPHY011941 | -8.27 |  | IMPHY000211 | -7.07 |
|  | IMPHY007909 | -8.24 |  | IMPHY003164 | -6.99 |
|  | IMPHY007737 | -8.19 |  | IMPHY001629 | -6.97 |
|  | IMPHY002477 | -8.1 |  | IMPHY004657 | -6.96 |
|  | IMPHY010508 | -8.07 |  | IMPHY007663 | -6.95 |
|  | IMPHY010989 | -8.05 |  | IMPHY000527 | -6.91 |
|  | IMPHY010321 | -8.04 |  | IMPHY000403 | -6.88 |
|  | IMPHY012556 | -8.04 |  | IMPHY006761 | -6.87 |
|  | IMPHY000661 | -8 |  | IMPHY001480 | -6.83 |
|  | IMPHY010466 | -8 |  | IMPHY001908 | -6.8 |
|  | IMPHY000331 | -8 |  | IMPHY003806 | -6.77 |
|  | FDA-56640146 | -8 |  | IMPHY003563 | -6.67 |
|  | IMPHY011940 | -7.99 |  | IMPHY000870 | -6.66 |
|  | IMPHY009003 | -7.98 |  | IMPHY014981 | -6.62 |
|  | IMPHY003155 | -7.97 |  | IMPHY005976 | -6.56 |
|  | IMPHY007525 | -7.94 |  | IMPHY008557 | -6.56 |
|  | IMPHY009364 | -7.93 |  | IMPHY009786 | -6.39 |
|  | IMPHY002751 | -7.92 |  | IMPHY011109 | -6.22 |
|  | IMPHY009438 | -7.9 |  | IMPHY013212 | -6.17 |
|  | IMPHY010711 | -7.9 |  | IMPHY007035 | -6.1 |
|  | IMPHY007735 | -7.89 |  | IMPHY002011 | -5.87 |
|  | IMPHY003936 | -7.88 |  | FDA-10445549 | -5.49 |
|  | IMPHY005785 | -7.87 |  | IMPHY004739 | -5.47 |
|  | IMPHY007736 | -7.86 |  | FDA-45375808 | -5.05 |
|  | IMPHY005188 | -7.86 |  | FDA-492405 | -4.31 |
|  | IMPHY006882 | -7.83 |  | FDA-37542 | -4.28 |
|  | IMPHY009090 | -7.76 |  | FDA-121304016 | -3.44 |
|  | IMPHY011458 | -7.71 |  |  |  |
