# Supplementary figures and images for: Exploring phytochemicals as potential pharmacological inhibitors for NS1 protein of Kyasanur forest disease virus using virtual screening, molecular docking, and molecular simulation approach
Source: PeerJ. 2025 Oct 9;13:e19954. doi: 10.7717/peerj.19954 (PMC12515432; doi:10.7717/peerj.19954)

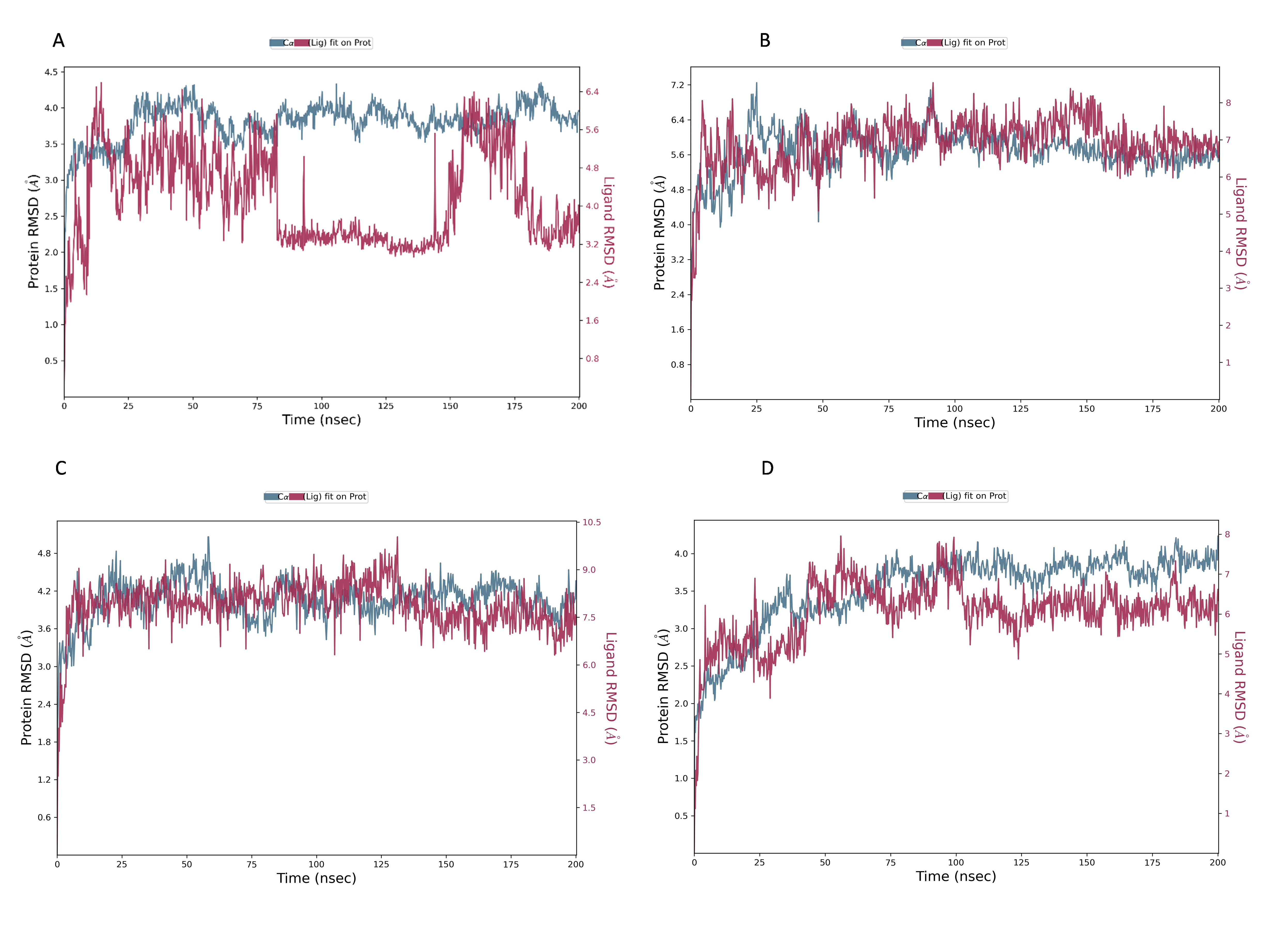

Supplement: Supplemental Information 4 [file peerj-13-19954-s004.png]

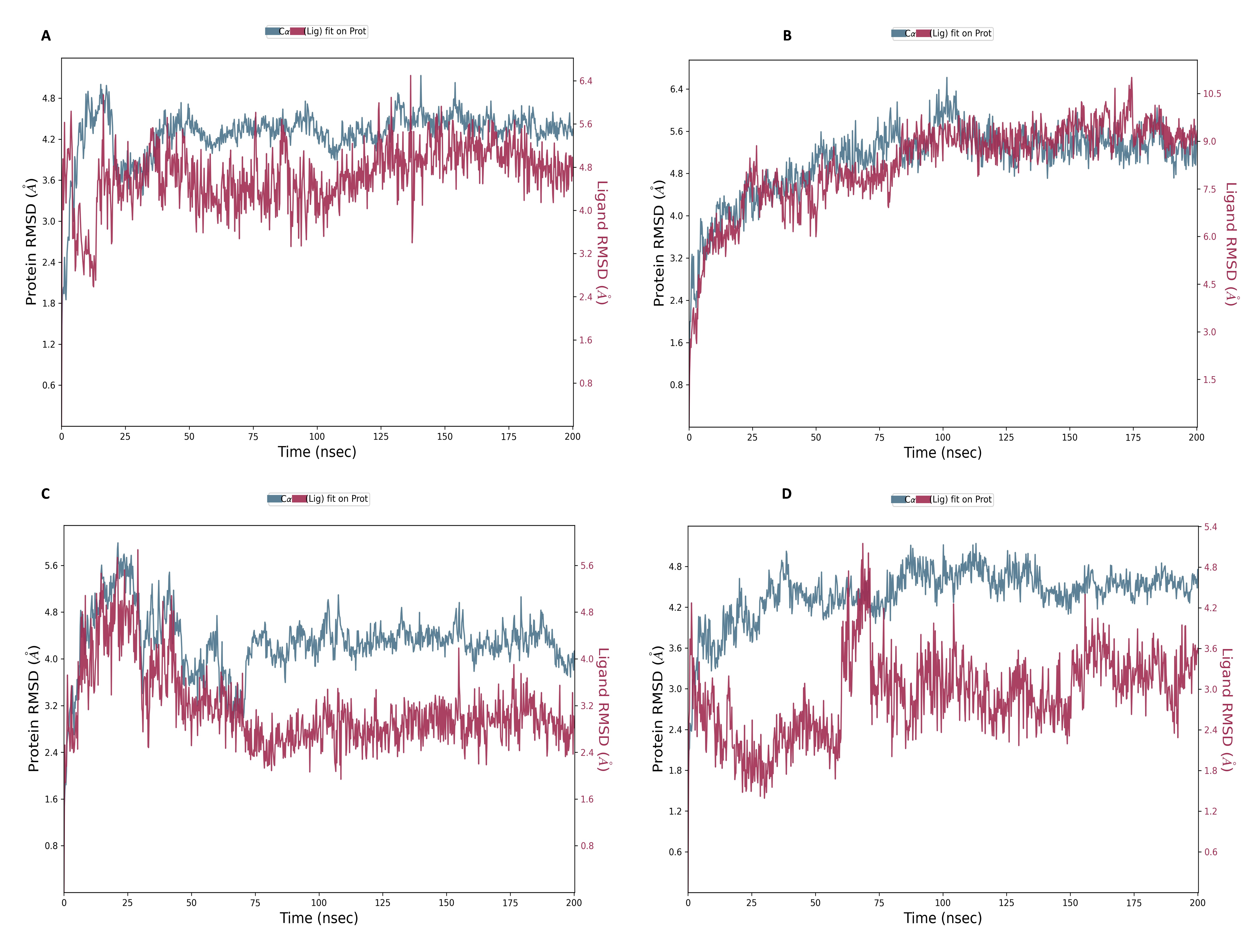

Supplement: Supplemental Information 5 [file peerj-13-19954-s005.png]

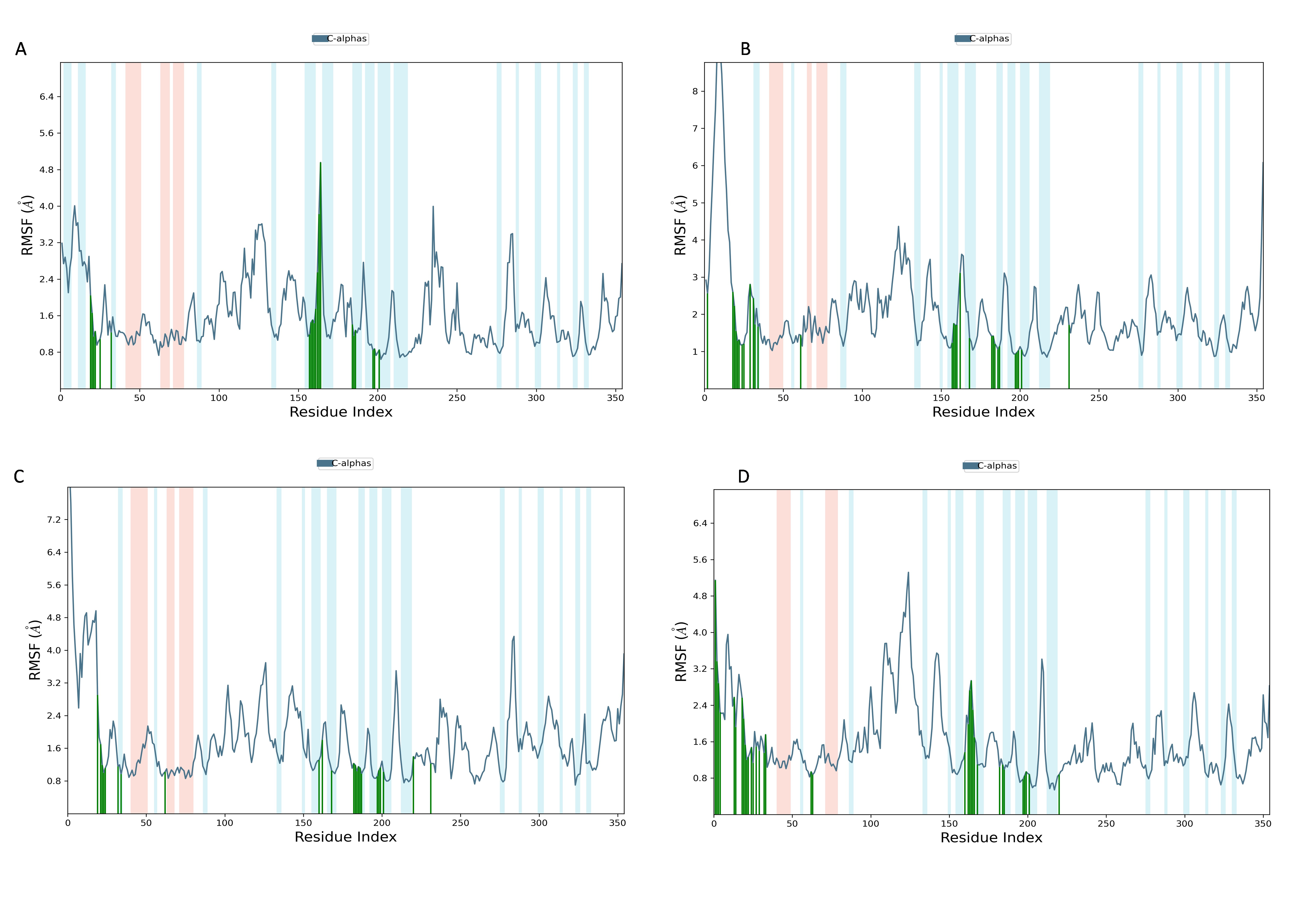

Supplement: Supplemental Information 6 [file peerj-13-19954-s006.png]

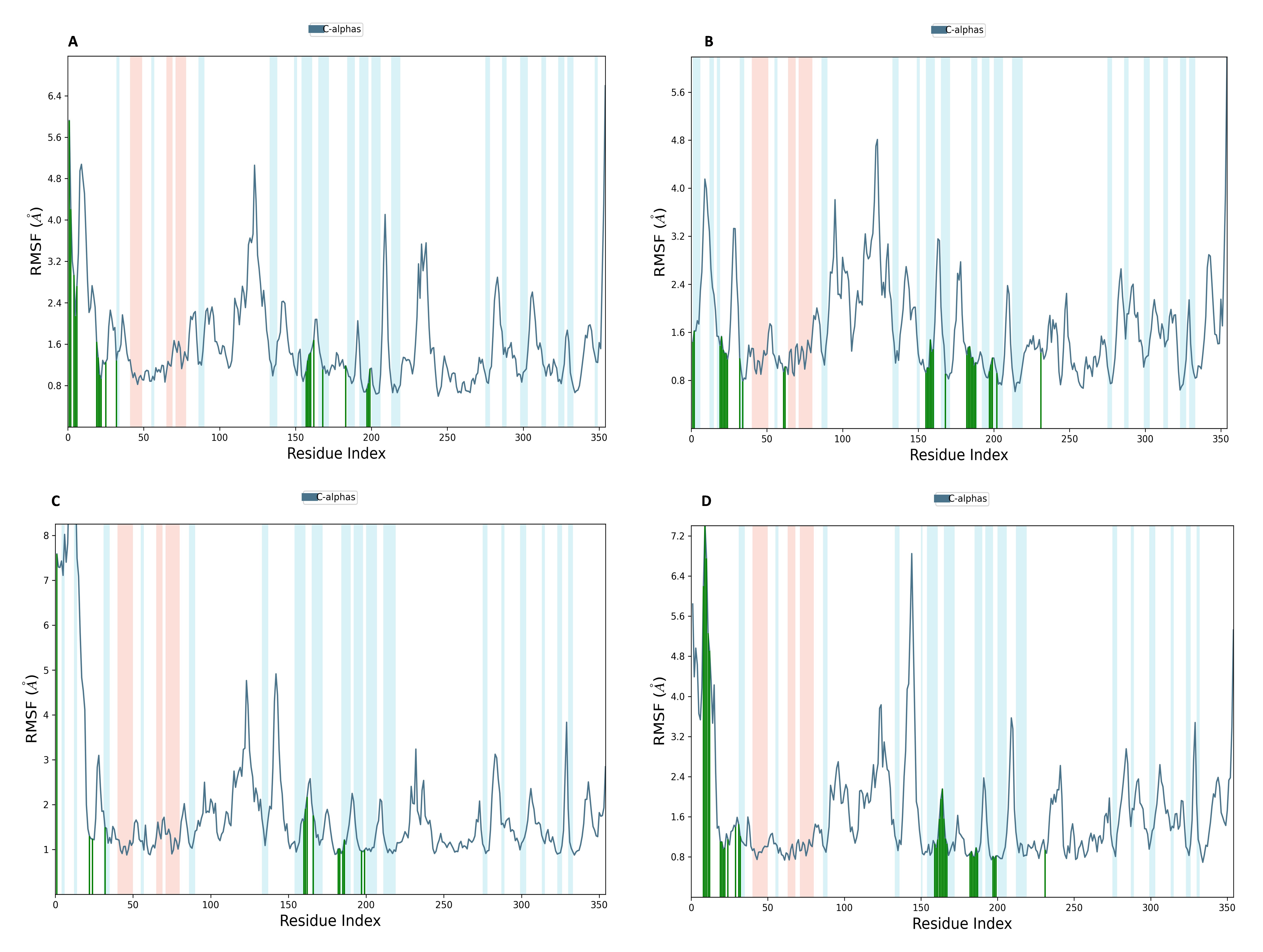

Supplement: Supplemental Information 7 — (A) NS1–L2 complex, (B) NS1–L3 complex, (C) NS1–L5 complex, and (D) NS1–dasabuvir complex. [file peerj-13-19954-s007.png]

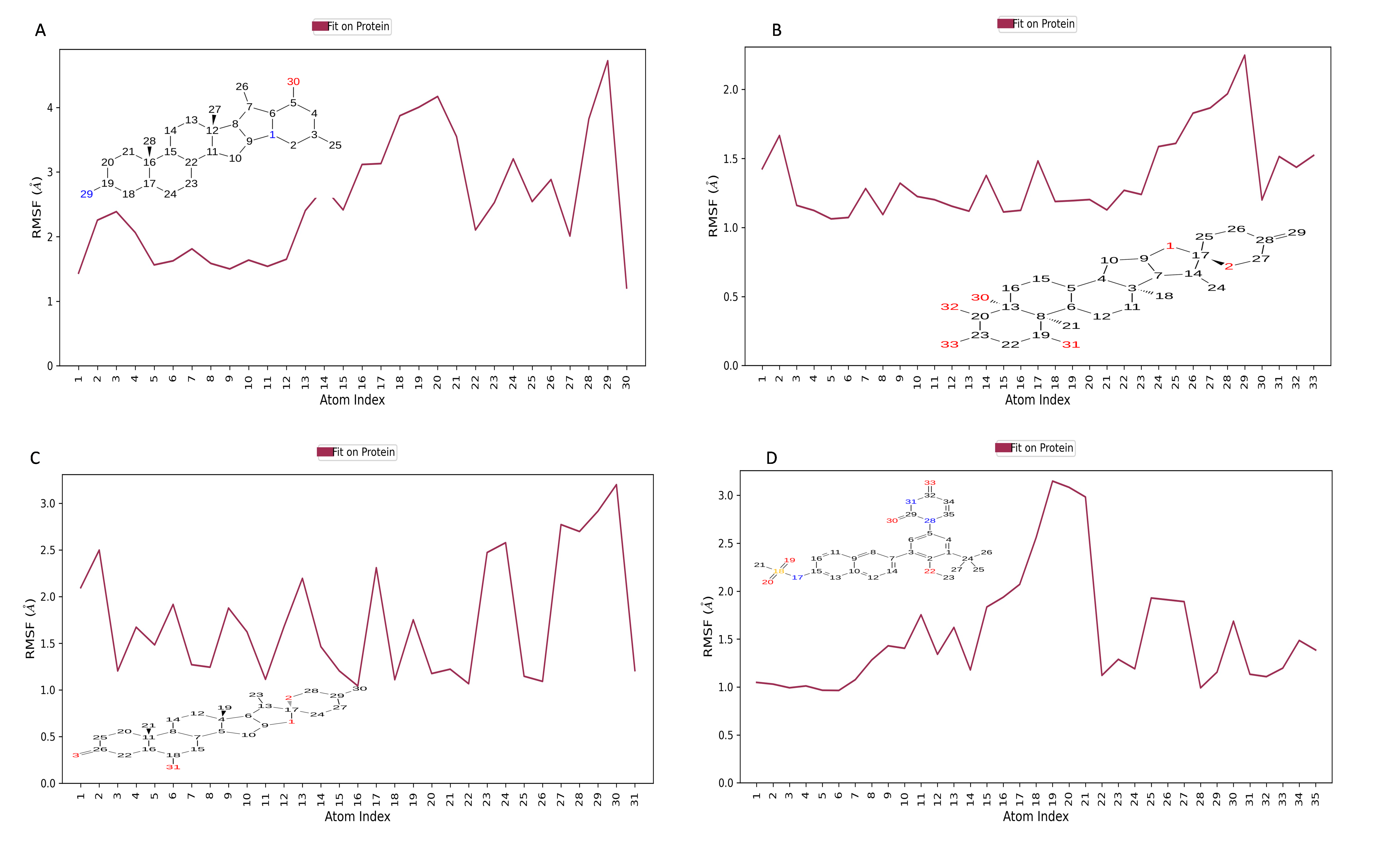

Supplement: Supplemental Information 8 [file peerj-13-19954-s008.png]

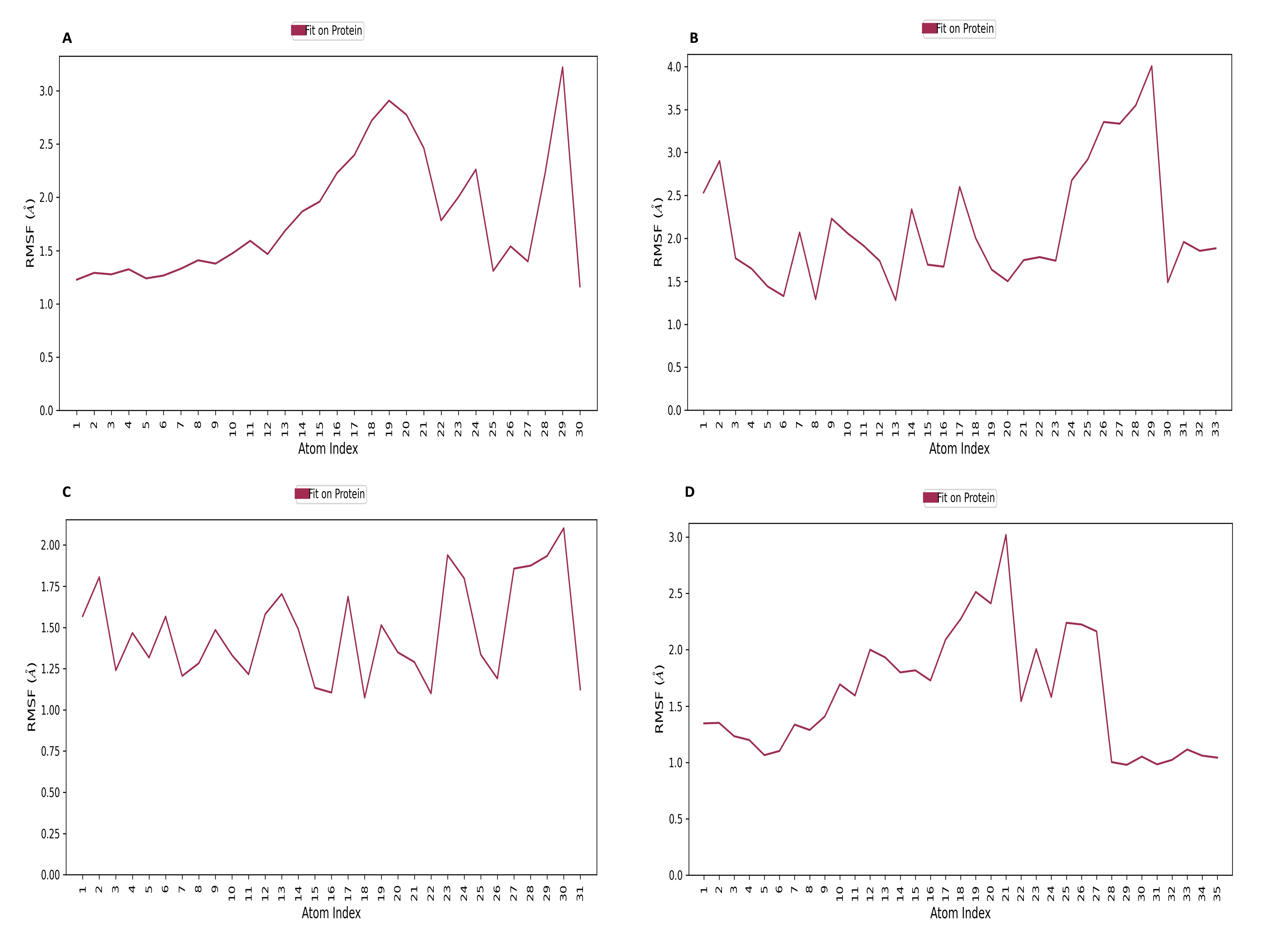

Supplement: Supplemental Information 9 — (A) NS1–L2 complex, (B) NS1–L3 complex, (C) NS1–L5 complex, and (D) NS1–dasabuvir complex. [file peerj-13-19954-s009.png]

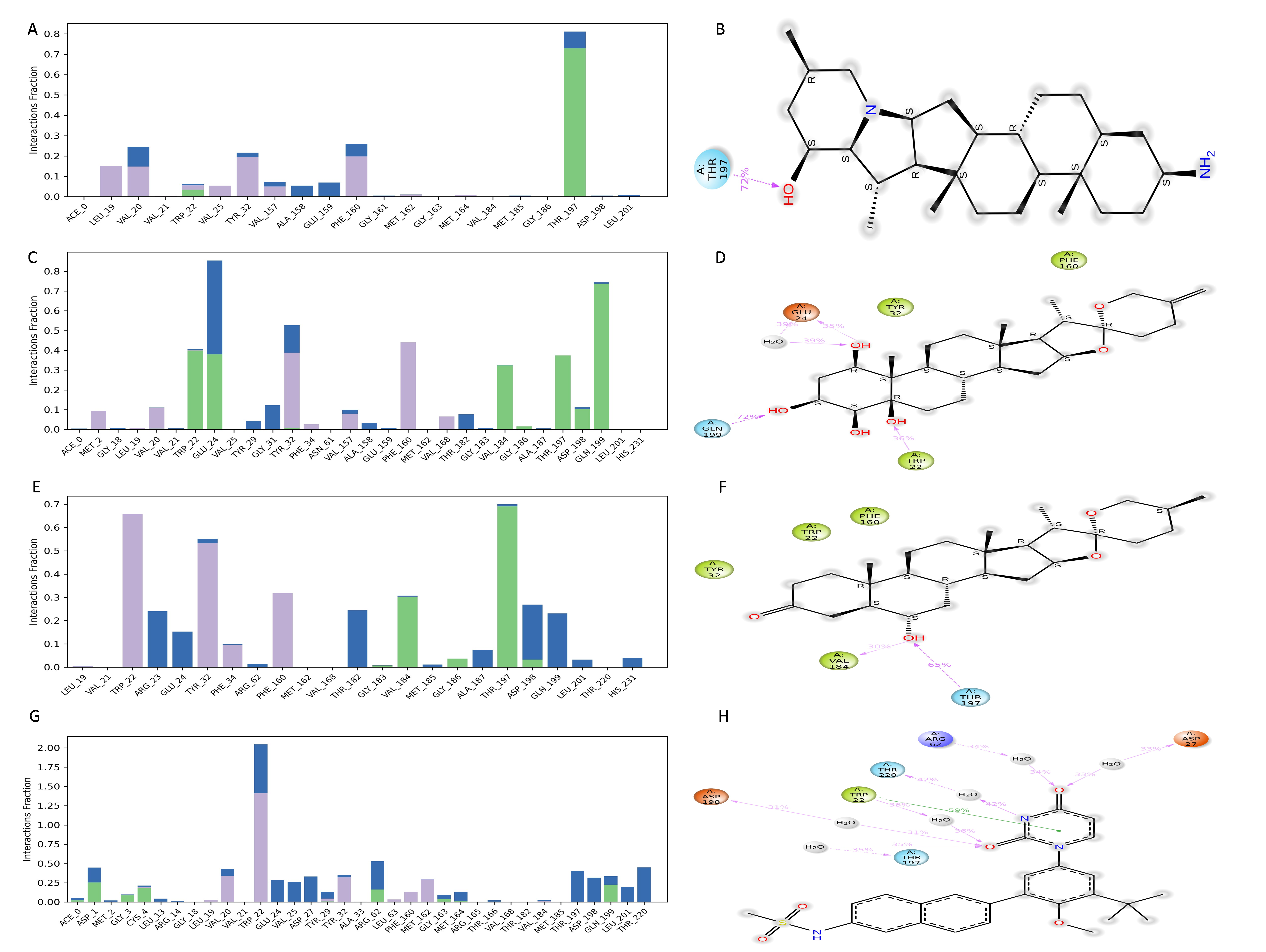

Supplement: Supplemental Information 10 [file peerj-13-19954-s010.png]

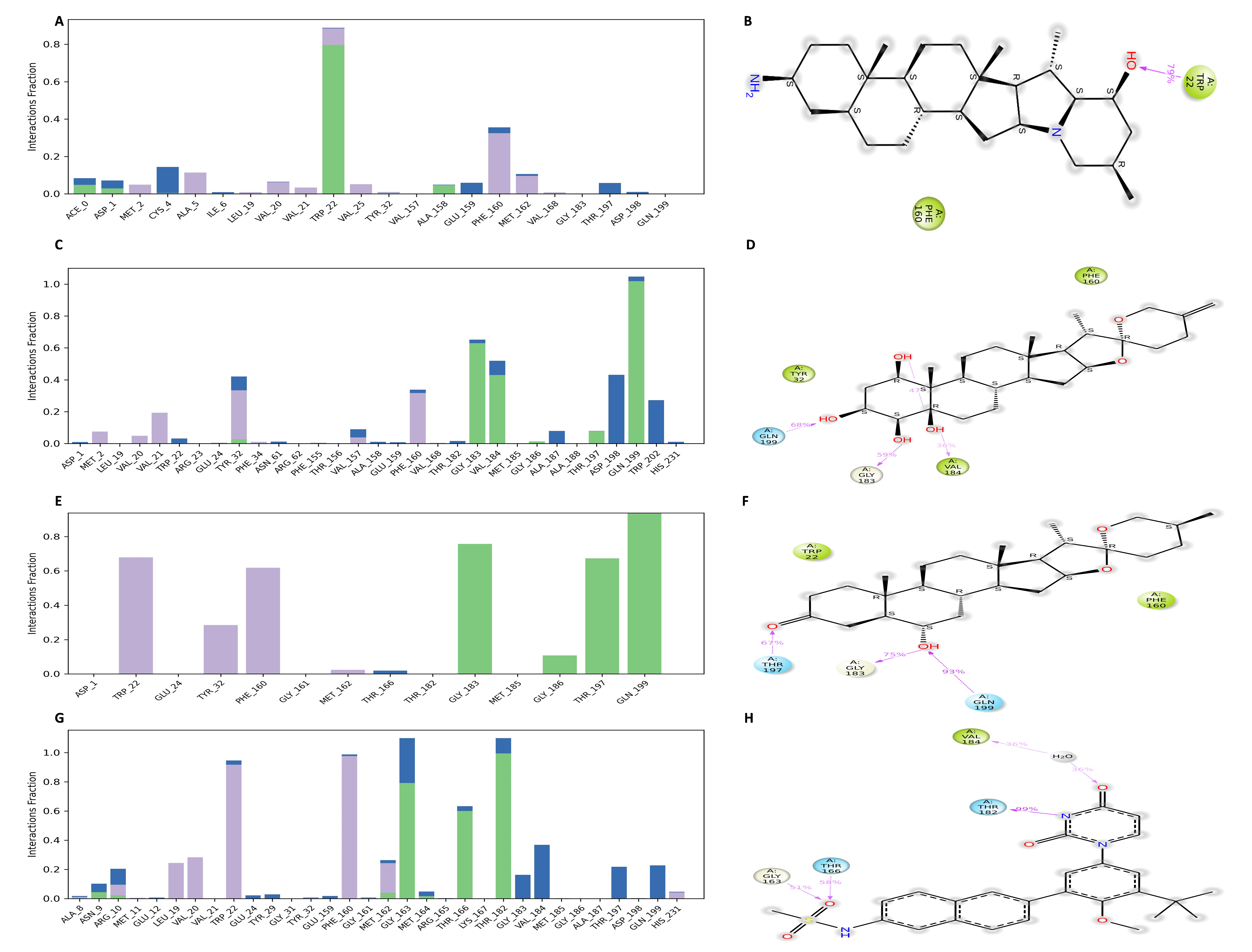

Supplement: Supplemental Information 11 — (A) NS1-L2 complex, (C) NS1-L3 complex, (E) NS1-L5 complex, & (G) NS1-dasabuvir complex and NS1-ligand contact for more than 30% simulation time (B) NS1-L2 complex, (D) NS1-L3 complex, (F) NS1-L5 complex, & (H) NS1-dasabuvir complex. [file peerj-13-19954-s011.png]

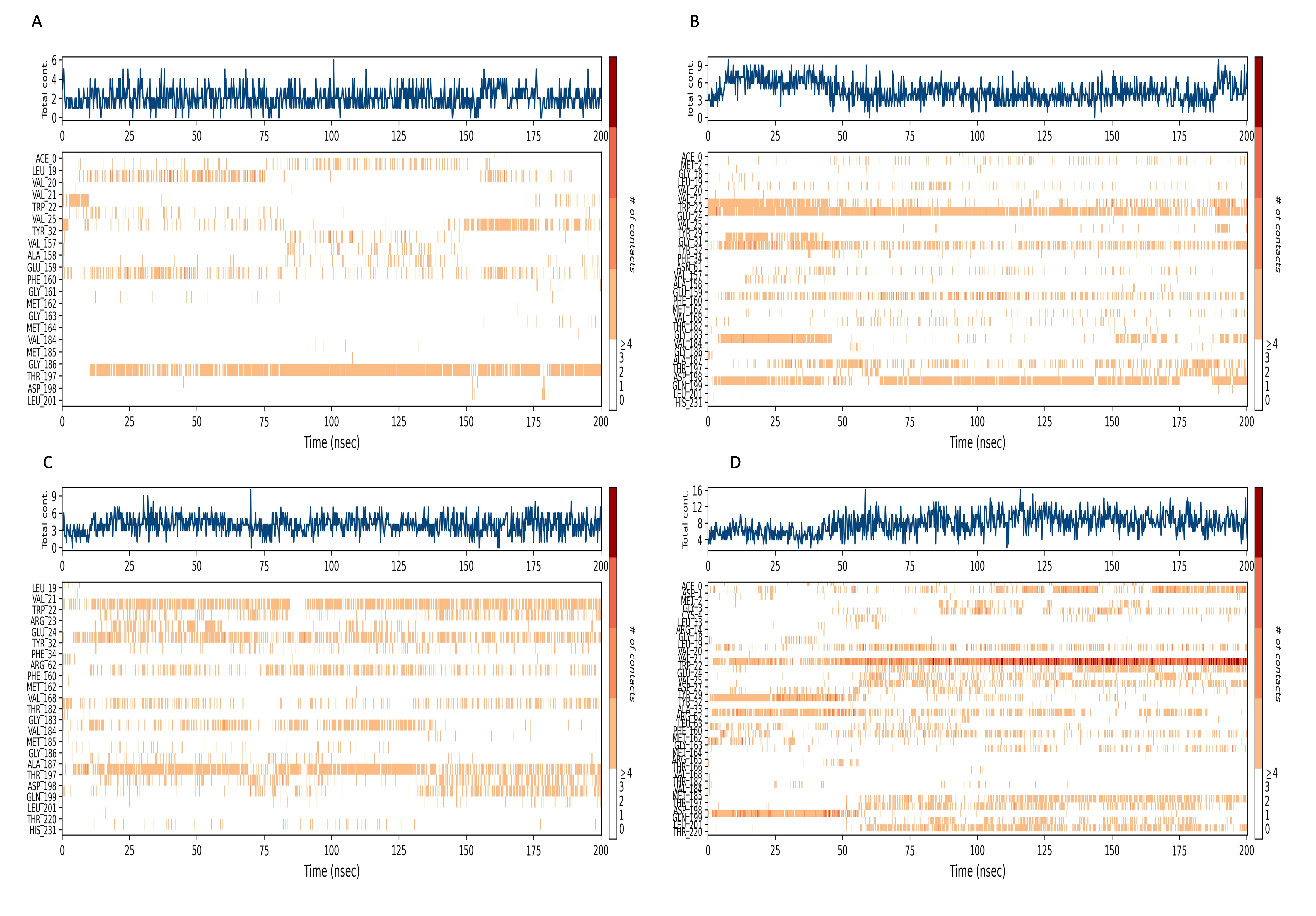

Supplement: Supplemental Information 12 [file peerj-13-19954-s012.png]

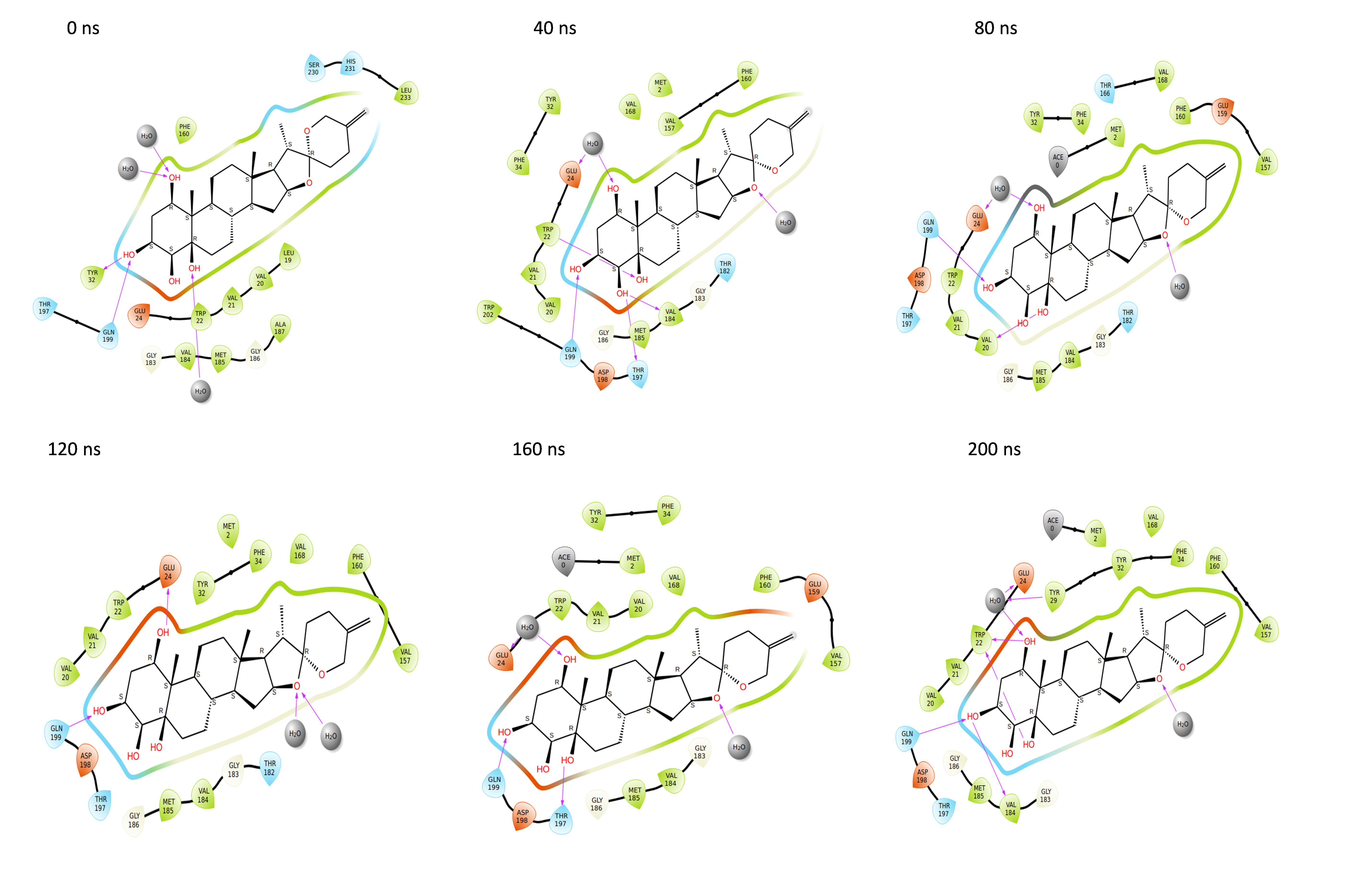

Supplement: Supplemental Information 13 [file peerj-13-19954-s013.png]

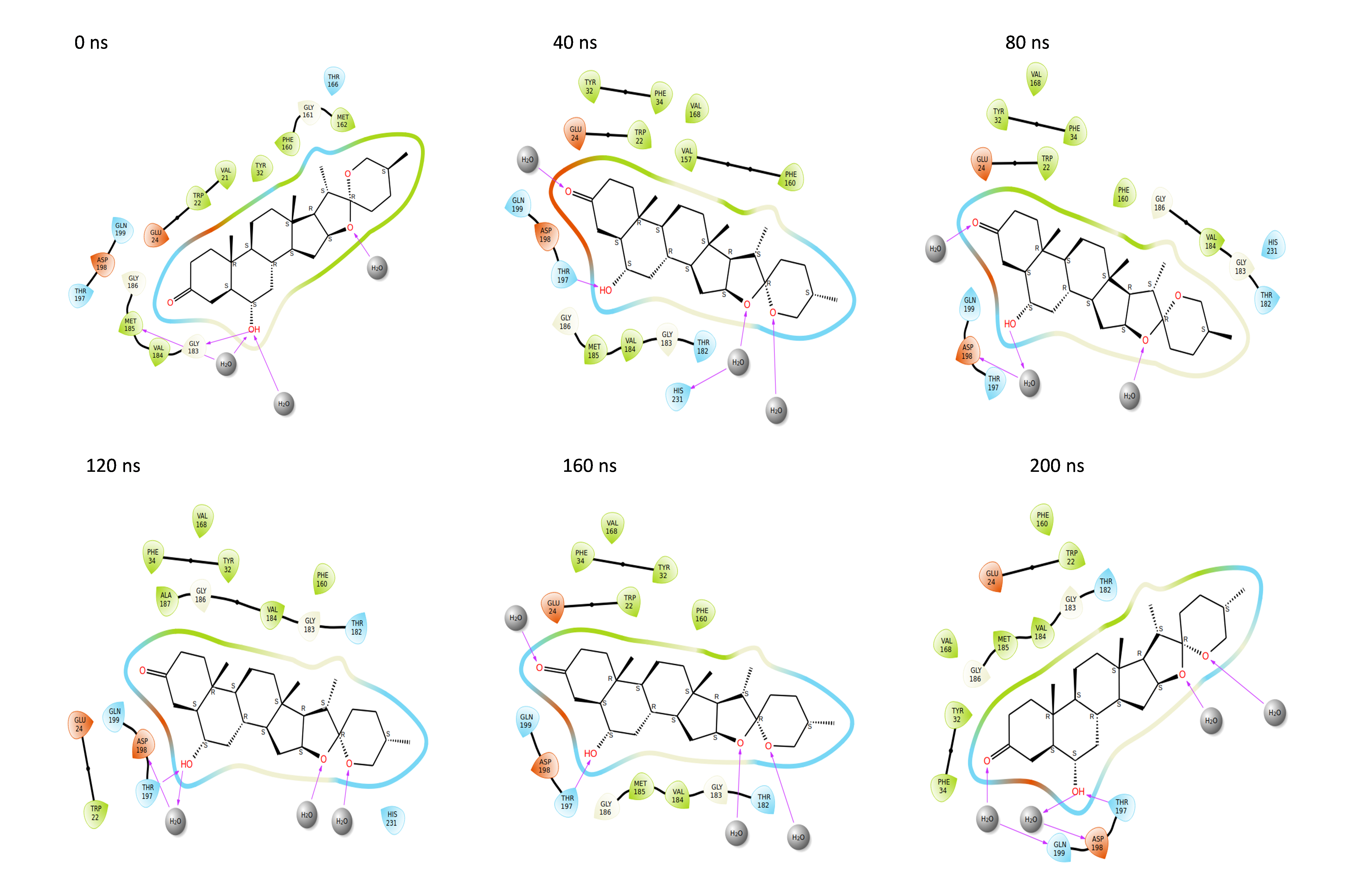

Supplement: Supplemental Information 14 [file peerj-13-19954-s014.png]

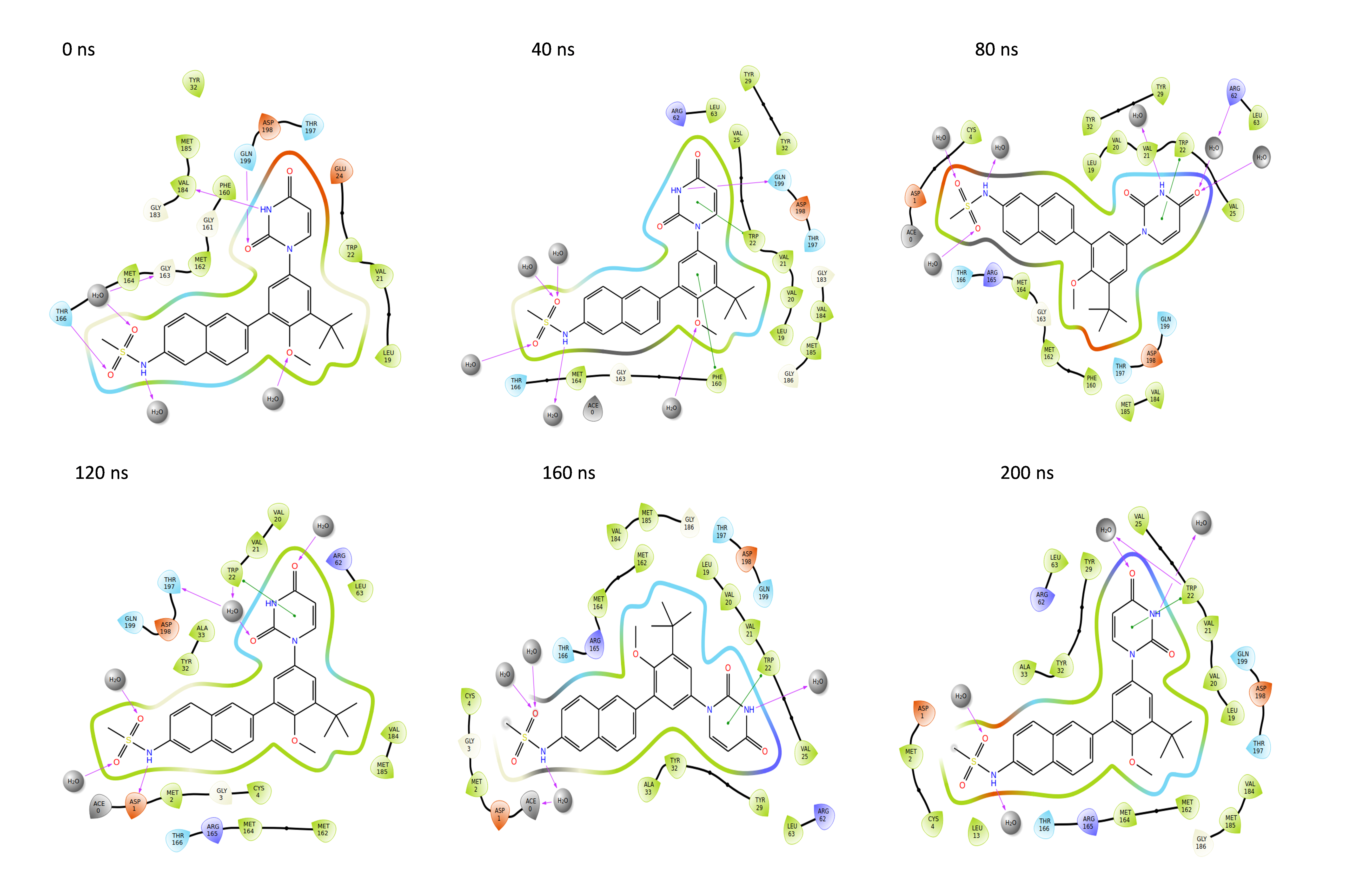

Supplement: Supplemental Information 15 [file peerj-13-19954-s015.png]

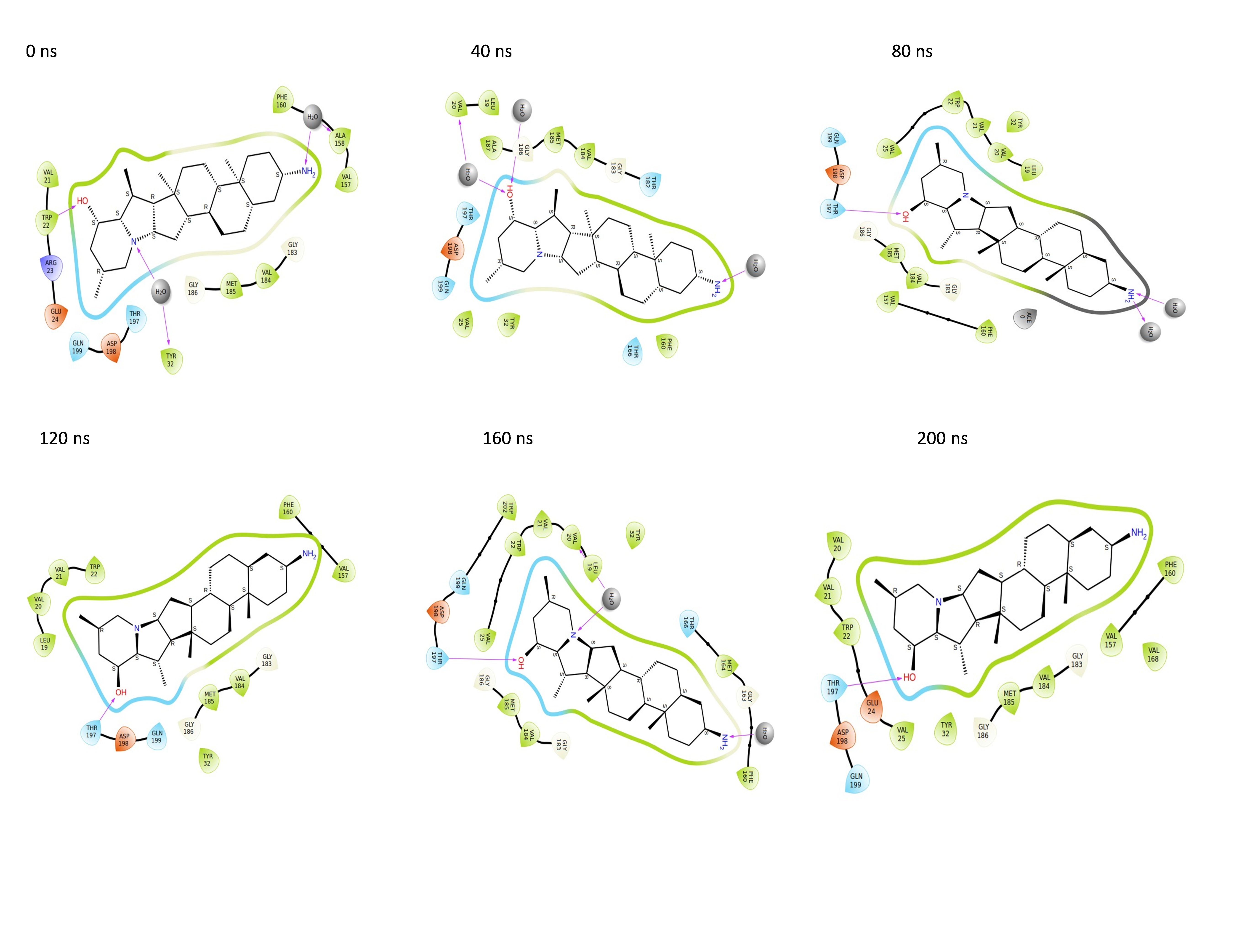

Supplement: Supplemental Information 16 [file peerj-13-19954-s016.png]

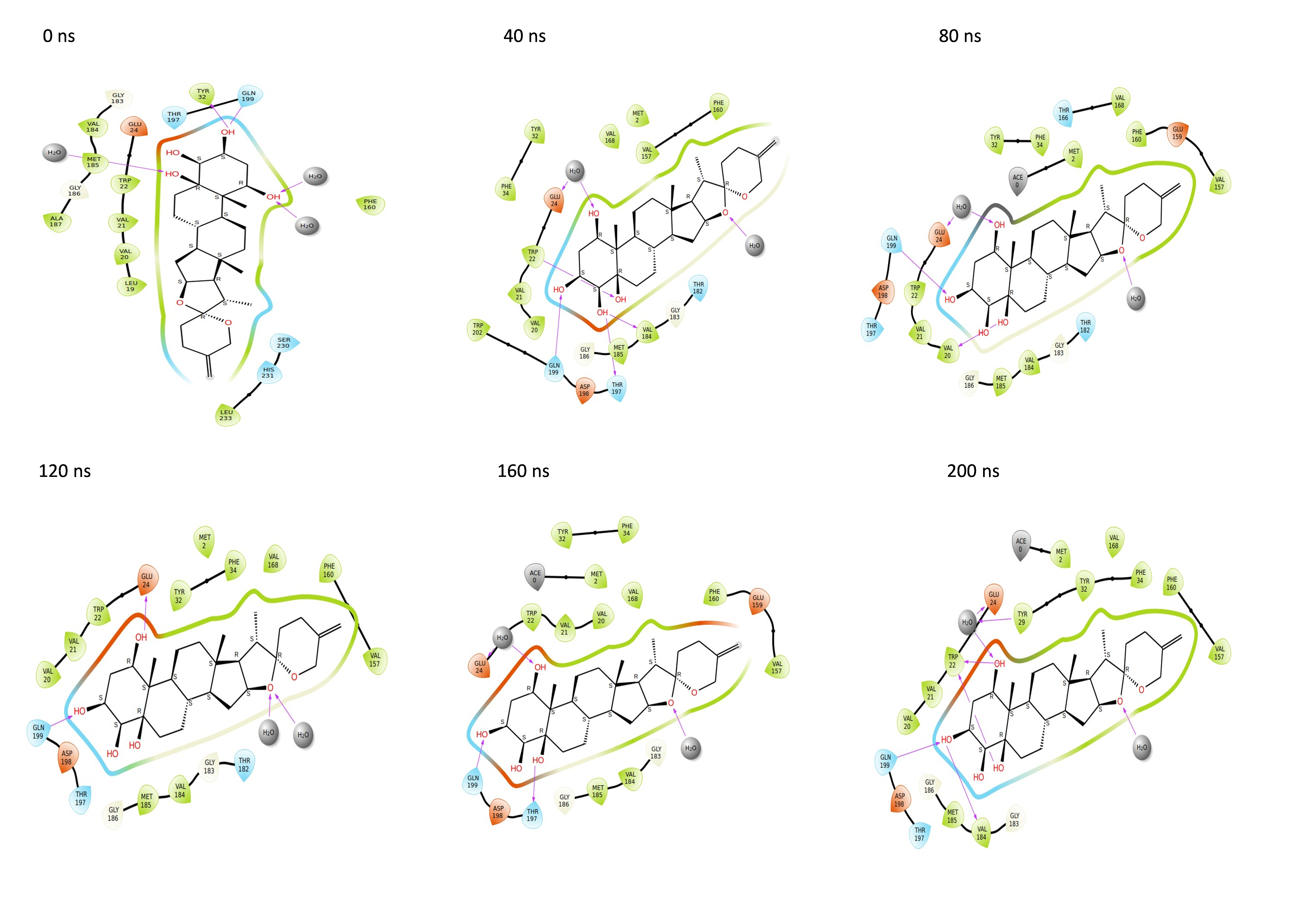

Supplement: Supplemental Information 17 [file peerj-13-19954-s017.png]

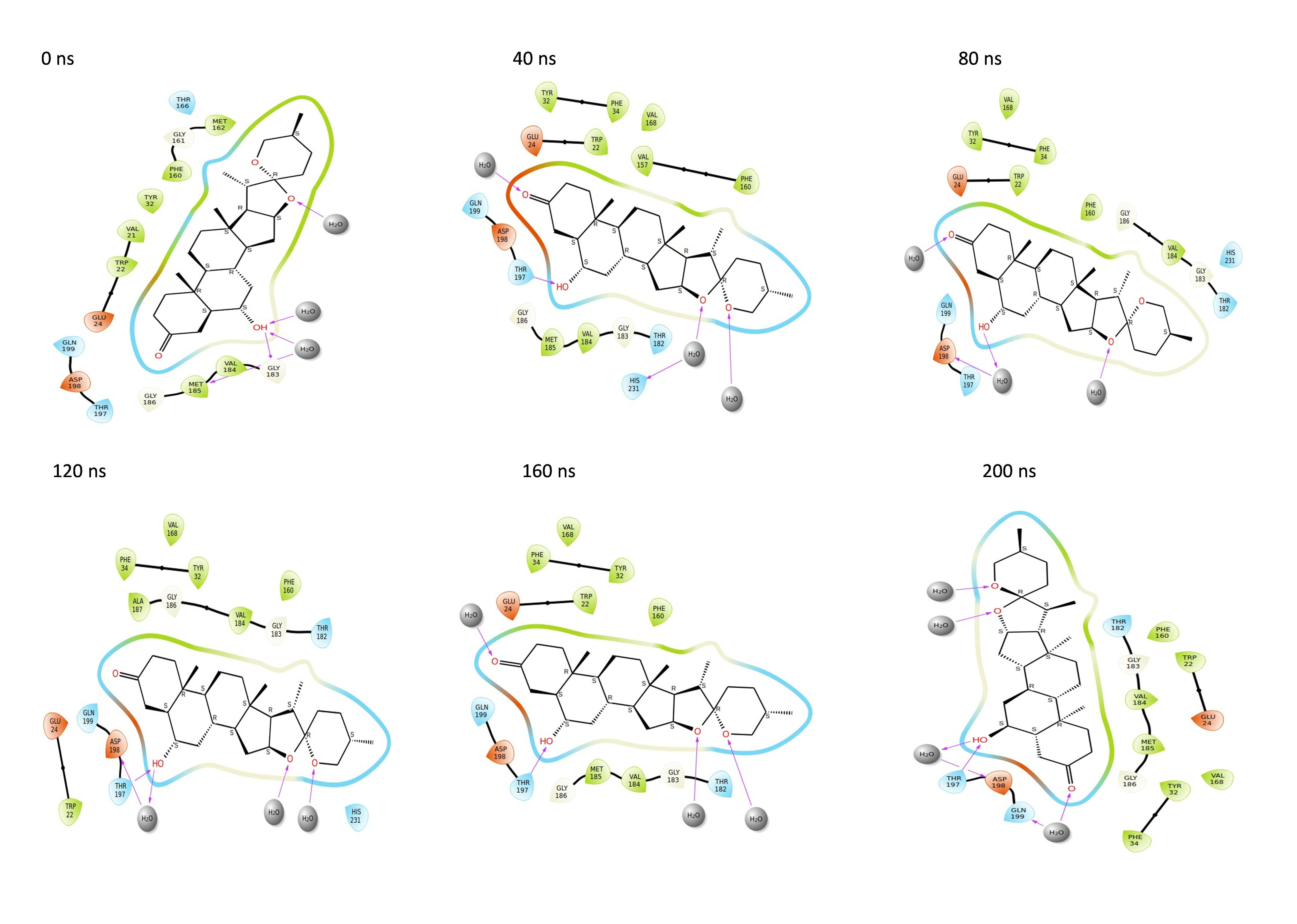

Supplement: Supplemental Information 18 [file peerj-13-19954-s018.png]

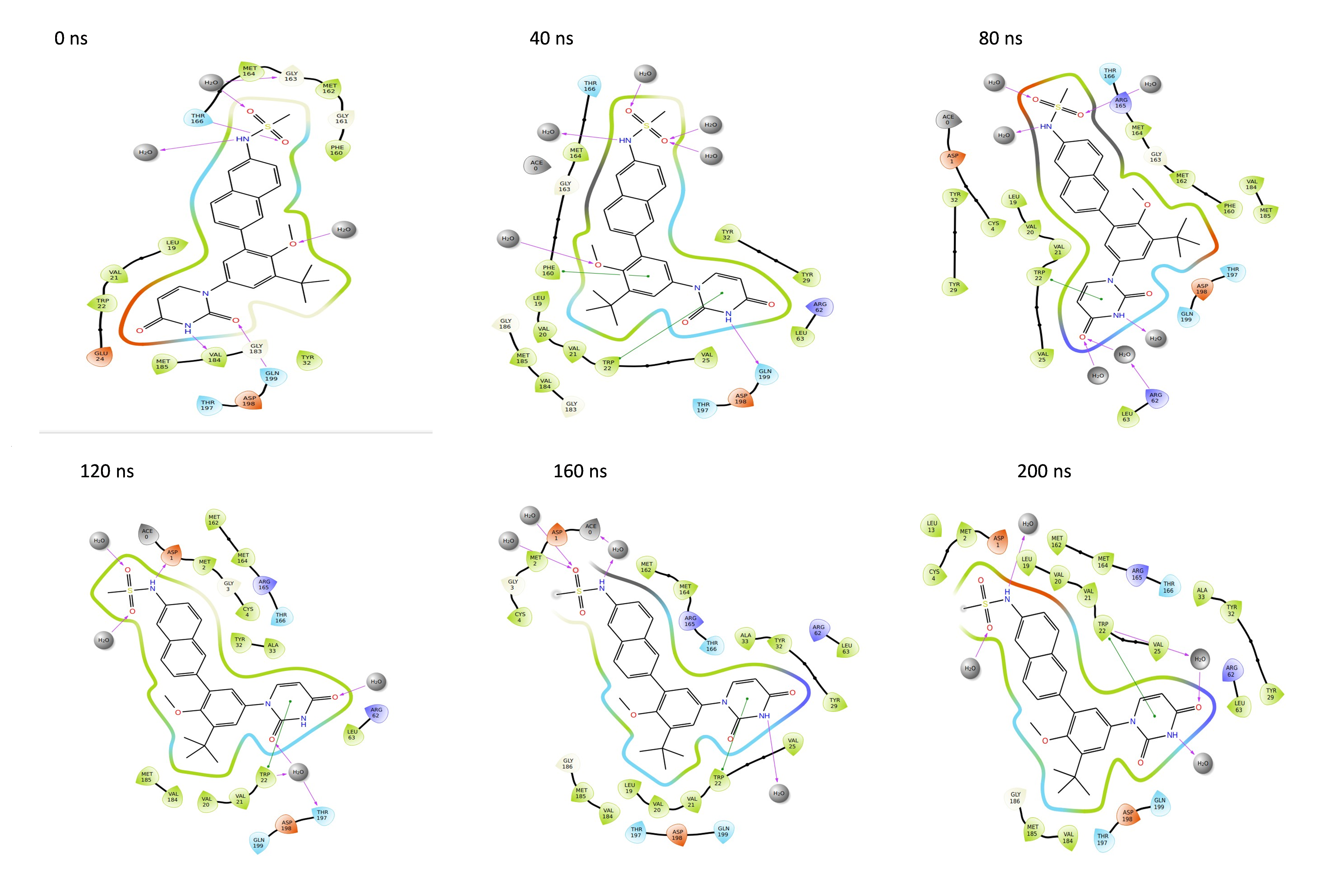

Supplement: Supplemental Information 19 [file peerj-13-19954-s019.png]
